# Circular RNA AKT3 upregulates PIK3R1 to enhance cisplatin resistance in gastric cancer via miR-198 suppression

**DOI:** 10.1186/s12943-019-0969-3

**Published:** 2019-03-30

**Authors:** Xiaoxu Huang, Zheng Li, Qiang Zhang, Weizhi Wang, Bowen Li, Lu Wang, Zhipeng Xu, Ailiang Zeng, Xing Zhang, Xuan Zhang, Zhongyuan He, Qiang Li, Guangli Sun, Sen Wang, Qing Li, Linjun Wang, Lu Zhang, Hao Xu, Zekuan Xu

**Affiliations:** 1 Department of General Surgery, The First Affiliated Hospital of Nanjing Medical University, 300 Guangzhou Road, Nanjing, 210029 Jiangsu province China; 2 Department of Gastrointestinal Surgery, The First Affiliated Yijishan Hospital of Wannan Medical College, Wuhu, Anhui China; 3 Department of Neurosurgery, The First Affiliated Hospital of Nanjing Medical University, Nanjing, China

**Keywords:** Cisplatin resistance, Gastric cancer, circAKT3, Circular RNA, miR-198, PIK3R1

## Abstract

**Background:**

Cisplatin (CDDP) treatment is one of the most predominant chemotherapeutic strategies for patients with gastric cancer (GC). A better understanding of the mechanisms of CDDP resistance can greatly improve therapeutic efficacy in patients with GC. Circular RNAs (circRNAs) are a class of noncoding RNAs whose functions are related to the pathogenesis of cancer, but, in CDDP resistance of GC remains unknown.

**Methods:**

circAKT3 (hsa_circ_0000199, a circRNA originating from exons 8, 9, 10, and 11 of the AKT3 gene) was identified by RNA sequencing and verified by quantitative reverse transcription PCR. The role of circAKT3 in CDDP resistance in GC was assessed both in vitro and in vivo. Luciferase reporter assay, biotin-coupled RNA pull-down and fluorescence in situ hybridization (FISH) were conducted to evaluate the interaction between circAKT3 and miR-198. Functional experiments were measured by western blotting, a cytotoxicity assay, clonogenic assay and flow cytometry.

**Results:**

The expression of circAKT3 was higher in CDDP-resistant GC tissues and cells than in CDDP-sensitive samples. The upregulation of circAKT3 in GC patients receiving CDDP therapy was significantly associated with aggressive characteristics and was an independent risk factor for disease-free survival (DFS). Our data indicated that circAKT3 promotes DNA damage repair and inhibits the apoptosis of GC cells in vivo and in vitro. Mechanistically, we verified that circAKT3 could promote PIK3R1 expression by sponging miR-198.

**Conclusions:**

circAKT3 plays an important role in the resistance of GC to CDDP. Thus, our results highlight the potential of circAKT3 as a therapeutic target for GC patients receiving CDDP therapy.

**Electronic supplementary material:**

The online version of this article (10.1186/s12943-019-0969-3) contains supplementary material, which is available to authorized users.

## Background

Gastric cancer (GC) is the most common malignant tumor of the digestive tract in East Asia and the third leading cause of cancer-related death worldwide [1, 2]. At present, the main treatments for advanced GC are systemic chemotherapy and palliative surgery, but the overall median survival after treatment is only 8 to 11 months [3]. In patients with histologically confirmed advanced GC and who are chemotherapy-naïve, cisplatin (CDDP) and fluorouracil-based chemotherapies were deemed as first-line treatments [4]. However, patients always acquired drug resistance after several cycles of CDDP-based treatment. Thus, chemotherapy resistance has limited overall clinical efficacy in patients [5, 6]. To improve GC patient survival, illuminating the molecular mechanism underlying CDDP resistance in GC is essential.

The cytotoxicity of CDDP is mediated by its interaction with DNA to form DNA adducts. Intracellular CDDP primarily binds to nuclear DNA with high affinity and can physically interact with mitochondrial DNA (mtDNA), which is involved in the activation of several signaling pathways and apoptosis [7–9]. In recent years, studies have shown that the PI3K/AKT signaling pathway could suppress cell apoptosis and facilitate cell survival. This PI3K/AKT signaling function is crucial in the regulation of chemotherapy resistance of cancer cells [10, 11]. Activated PI3K/AKT signaling promotes the phosphorylation of caspase-3 and prevents the activation of caspase-3 and the inhibition of apoptosis [12].

Circular RNAs (circRNAs), a category of noncoding RNAs (ncRNAs), play a crucial role in the process of transcriptional and posttranscriptional gene expression [13]. Recently, circRNAs were found to function as competitive endogenous RNAs (ceRNAs) to sponge microRNAs (miRNAs) and then suppress their functions, indicating a novel mechanism for regulating miRNA activity and providing a promising mode of action for circRNAs. As miRNAs regulate a series of biological processes, circRNA sponge activity will affect these biological behaviors as well [13]. miRNAs are a large class of short (~ 22 nt) ncRNAs that posttranscriptionally regulate gene expression through direct base pairing to target sites within mRNAs. circRNAs can affect miRNA activities by competing for miRNA-binding sites [13]. However, the function of circRNAs as miRNA sponges has not been clearly elucidated in GC resistance to CDDP.

To investigate the potential roles of circRNAs in the regulation of CDDP resistance in GC, we performed RNA sequencing (RNA-Seq) and verified thousands of distinct circRNAs in CDDP-sensitive and CDDP-resistant GC cells from humans. Through functional gain and loss experiments, we further observed that hsa_circ_0000199, which originates from exons 8, 9, 10, and 11 of the AKT3 gene and is termed circAKT3, was significantly upregulated in both CDDP-resistant GC tissues and CDDP-resistant cells. Furthermore, we found that circAKT3 modulates CDDP sensitivity by sponging miR-198 that suppresses PIK3R1 expression via activation of the PI3K/AKT pathway in GC.

## Methods

### Patients and samples

In total, 149 GC tissues (cohorts 1, 2) were obtained from the First Affiliated Hospital of Nanjing Medical University. All samples were collected in accordance with HIPAA guidelines and approved institutional protocols. Patients received treatment with standard CDDP-based therapeutic regimens after surgery. Disease-free survival (DFS) was defined as the time interval between gastrectomy (R0 excision) and the time of either disease recurrence or disease-associated death. CDDP resistance was defined as tumor relapse during CDDP-based chemotherapy after R0 excision, and CDDP sensitivity was defined as no tumor recurrence during CDDP-based therapy; both definitions followed standard CDDP response definitions published elsewhere [14]. Forty-four samples (Cohort 1) were used for circRNAs validation, and another 105 samples (Cohort 2) were used to quantify circAKT3 levels and to analyze the correlation between circAKT3 expression and outcomes after R0 excision in patients undergoing CDDP-based chemotherapy. The samples from cohorts 1 and 2 were obtained in 2013–2016 and 2007–2011, respectively. The grouping of the ROC curve was based on the median relative expression of circAKT3. Detailed information is listed in Additional file 1: Table S1.

### Cell culture

The CDDP-sensitive cell lines SGC7901 and BGC823 as well as their CDDP-resistant strains (SGC7901CDDP and BGC823CDDP, respectively) were maintained in RPMI 1640 medium (Wisent, Shanghai, China) supplemented with 10% fetal bovine serum (FBS) (Wisent, Biocenter, China) (Additional file 2: Figure S1A). 293 T cells were cultured in DMEM with high glucose (Gibco-BRL, Carlsbad, CA, USA) supplemented with 10% FBS. 293 T, SGC7901CDDP, BGC823 and SGC7901 cells were purchased from the Cell Bank of Type Culture Collection of Chinese Academy of Sciences, and BGC823CDDP cells were established as previously described [15].

### miRNA targets prediction of circAKT3

To predict the miRNA-binding sites of circAKT3, we used the bioinformatic databases miRanda, PITA and RNAhybrid. Filtering restrictions were as follows: (1) total score ≥ 140, total energy < 17 kcal/mol; (2) combined interaction energy (△△G) < 10; and (3) minimum free energy (MFE) ≤ 20 kcal/mol. Detailed information is listed in Additional 3: Dataset S1.

### RNA preparation, treatment with RNase R, and PCR

Total RNA was extracted from GC cells or tissues using TRIzol Reagent (Invitrogen, 15,596,018). RNase R treatment was carried out for 15 min at 37 °C using 3 U/mg RNase R (Epicenter). For Quantitative real-time PCR (RT-PCR), 500 ng of treated RNA was directly reverse transcribed using Prime Script RT Master Mix (Takara, Japan) and either random or oligo(dT) primers. Reverse transcription of miRNA was performed using a New Poly(A) Tailing Kit (ThermoFisher Scientific, China). mRNA was reverse transcribed into cDNA with a PrimeScript RT Master Mix Kit (Takara, RR036A, Japan). cDNA was amplified using Universal SYBR Green Master Mix (4,913,914,001, Roche, Shanghai, China). The CT value was measured during the exponential growth phase. Relative gene expression levels were determined using the 2^-△△CT^ method. The primers used are listed in Additional file 1: Table S2.

### Isolation of nuclear and cytoplasmic fractions

SGC7901CDDP and BGC823CDDP cells were lysed on ice for 10 min in 0.3% NP-40/NIB-250 buffer (15 mM Tris–HCl (pH 7.5), 60 mM KCl, 15 mM NaCl, 5 mM MgCl2, 1 mM CaCl_2_ and 250 mM sucrose) supplemented with protease inhibitors. After centrifugation at 600 × g for 5 min at 4 °C, the resultant supernatant was collected as the cytoplasmic fraction and mixed with an equal volume of TRIsure reagent. After the pellet was washed with NIB-250, the nuclei were lysed in TRIsure reagent.

### Vector construction

The method for overexpressing circRNAs was reported previously [16]. For the construction of circAKT3 overexpression plasmids, human circAKT3 cDNA was amplified using PrimerSTAR Max DNA Polymerase Mix (Takara, RR036A, Japan) and inserted into the pCD5-ciR vector (Greenseed Biotech Co, Guangzhou, China). The pCD5-ciR vector contains a front circular frame and a back circular frame. Transfection was carried out using Lipofectamine 2000 (Invitrogen) according to the manufacturer’s instructions. The luciferase reporter containing the circAKT3 sequence in the 3′-UTR was constructed by subcloning the circAKT3 fragment into the region directly downstream of a cytomegalovirus promoter-driven firefly luciferase (FL) cassette in a pCDNA3.0 vector. Mutations of each miRNA-binding site in the circAKT3 sequence were created using a Mut Express II Fast Mutagenesis Kit (Vazyme, Nanjing, China). The mutations were introduced in both the circAKT3-expressing vector and the luciferase reporter containing the circAKT3 sequence.

### Oligonucleotide transfection

siRNA and miRNA mimics and inhibitors were synthesized by GenePharma (Shanghai, China). The sequences used are listed in Additional file 1: Tables S3 and S4. Transfection was carried out using Lipofectamine RNAiMAX (Life Technologies) according to the manufacturer’s instructions.

### RNA pull-down

A pull-down assay was performed as described previously [17, 18]. The biotin-labeled circAKT3 probe was synthesized by RiboBio (Guangzhou, China). In brief, 1 × 10^7^ circAKT3-overexpressing GC cells were harvested, lysed, and sonicated. The circAKT3 or oligo probe was incubated with streptavidin-coupled Dynabeads (Invitrogen) at 30 °C overnight to generate probe-bound Dynabeads. After the treated beads were washed with wash buffer, the RNA complexes bound to the beads were eluted and disrupted with lysis buffer and proteinase K prior to RT-PCR or RT-qPCR. Biotinylated probes sequences used in this study (see Additional file 1: Table S5).

### Luciferase reporter assay

293 T, SGC7901CDDP and BGC823CDDP cells were seeded in 24-well plates and cotransfected with corresponding plasmids and miRNA mimics in triplicate. At 48 h after transfection, luciferase reporter assays were conducted using a dual-luciferase reporter assay system (Promega, Madison, WI) according to the manufacturer’s instructions. Relative luciferase activity was normalized to Renilla luciferase activity.

### Fluorescence in situ hybridization (FISH)

The double FISH assay was performed in SGC7901CDDP cells and GC tissues as previously described [16, 19]. Biotin-labeled probes specific to circAKT3 and Dig-labeled locked nucleic acid miR-198 probes were used in the hybridization (Exiqon, Vedbaek, Denmark). The sequences are listed in Additional file 1: Table S6, FISH probes sequences used in this study. The signals of the biotin-labeled probes were detected using Cy5-conjugated streptavidin (Life Technologies), and the signals of the Dig-labeled miR-198 probes were detected using a tyramide-conjugated Alexa 488 fluorochrome TSA kit. Nuclei were counterstained with 4,6-diamidino-2-phenylindole. Images were acquired on a Leica TCS SP2 AOBS confocal microscope (Leica Microsystems, Mannheim, Germany). CircAKT3 and miR-198 expression levels were evaluated by the proportions and intensities of the positive cells detected within 5 fields of view on every slide (400-fold magnification). Proportion scores were assigned as follows: < 10% = 0, 10–25% = 1, 26–50% = 2, 51–75% = 3 and > 75% = 4. Intensity scores were assigned as follows: 0 = no staining, 1 = weak, 2 = moderate, 3 = strong and 4 = significantly strong.

### Western blot analysis

For western blot analysis, cells were extracted using a protein extraction kit (Key Gene, KGP9100). Lipid proteins were added into 8, 10, 12% or 15% gels, subjected to 120 V to promote migration, and then transferred onto nitrocellulose membranes. The membranes were blocked with 5% BSA in TBST buffer and incubated with specific primary antibodies at 4 °C overnight. The next day, membranes were washed 3 times for 15 min in TBST and incubated with secondary antibodies for 2 h at room temperature. HRP substrate (WBKL0100, Millipore, USA) was used to detect the protein bands (Molecular Imager, ChemiDoc XRS+, BIO-RAD, USA), and the band intensities were quantified using Image-Pro Plus software (Mediacy, USA). Detailed information of antibody used in this study (see Additional file 1: Table S7).

### Cytotoxicity assay

The cytotoxicity assay was performed as previously described [15]. Cell viability was measured using Cell Counting Kit-8(CCK8)following the manufacturer’s directions (Dojindo, Kumamoto, Japan).

### Clonogenic assay

A clonogenic assay was performed as previously described [15]. At 48 h after transfection, BGC823CDDP, SGC7901CDDP, BGC823 and SGC7901 cells were cultured with CDDP at the indicated concentrations for 3 h. Then, the cells were harvested, seeded into six-well plates (500 cells per well) and cultured for an additional 2 (BGC823CDDP and SGC7901CDDP cells) or 3 weeks (BGC823 and SGC7901 cells). For scoring the colony-forming units, we fixed cells in 1 ml of methanol for 10 min and then stained the cells with crystal violet for 15 min.

### Apoptosis assay

Cell apoptosis was detected using a PI/Annexin V-FITC Apoptosis Detection Kit (BD Pharmingen, 556,547) according to the manufacturer’s instructions. Briefly, after GC cells were treated with CDDP at the indicated concentrations for 48 h in 6-well plates, they were harvested and resuspended in 300 ml of binding buffer. Next, 5 μl of Annexin V-FITC and 5 μl of PI were added to the suspensions, and the cells were incubated in the dark at 4 °C for 15 min. The samples were subsequently analyzed with a flow cytometer (Gallios, Beckman, USA).

### Actinomycin D assay

The Actinomycin D assay was performed as previously described [16]. SGC7901CDDP and BGC823CDDP cells were seeded in 5 wells in 24-well plates (5 × 10^4^ cells per well). Twenty-four hours later, the cells were exposed to Actinomycin D (2 μg/ml, Abcam, ab141058) for 0 h, 6 h, 12 h, 18 h and 24 h. The cells were then harvested, and the relative RNA levels of circAKT3 and AKT3 mRNA were analyzed by RT-qPCR and normalized to the values measured in the group in the 0 h group (mock treatment).

### Immunofluorescence staining

Cells seeded onto coated cover slips growth for 24 h, then treated with CDDP, and harvested the cells at 0, 2, and 8 h. The cells were fixed with 4% paraformaldehyde at room temperature for 15 min and then permeabilized with PBS containing 0.25% Triton X-100 for 10 min. Next, the cells were blocked with 1% BSA for 20 min before incubation with primary antibodies at room temperature for 2 h. After the cells were washed with PBS, they were incubated with appropriate secondary antibodies (FITC-conjugated goat anti-rabbit, Molecular Probes, USA) at room temperature for 2 h. Following a final wash with PBS, cells were mounted with antifading mounting medium containing DAPI. The images were captured with a Leica DMI3000B (Germany) fluorescence microscope.

### Transduction with lentivirus

SGC7901CDDP cells stably expressing circAKT3 siRNA (si-circ-1) and its negative control siRNA (si-NC) were generated by infection with lentiviruses as previously described [20]. Transfection was carried out according to the manufacturer’s instructions. The lentiviral expressing vectors were purchased from HanBio Co. Ltd. (Shanghai, China).

### Nude mouse xenograft model

Six-week-old female BALB/c nude mice were purchased from the Laboratory Animal Center of Nanjing Medical University and maintained under pathogen-free conditions. A total of 5 × 10^6^ SGC7901CDDP cells infected with lentivirus containing si-circ-1 or si-NC (2 μl of 10^9^ viral genomes μl^− 1^, HanBio) in 100 μl of PBS were subcutaneously injected into different sides of the groin of each mouse. One week after injection, we intraperitoneally injected mice with cisplatin (5 mg/kg) in PBS or PBS alone three times per week. The xenograft tumors were harvested after 5 weeks. The entire experimental protocol was conducted in accordance with the guidelines of the local institutional animal care and use committee.

### Immunohistochemical staining (IHC)

Xenografts and GC tissues exposed to the indicated concentrations of CDDP were prepared for IHC as previously described [21]. Sections were identified by IHC Imager (DM4000B, LEIKA, Germany), and target protein expression levels were evaluated by the proportions and intensities of positive cells detected within 5 fields of view on every slide (400-fold magnification). Proportion scores were assigned as follows: < 10% = 0, 10–25% = 1, 26–50% = 2, 51–75% = 3 and > 75% = 4. Intensity scores were assigned as follows: 0 = no staining, 1 = weak, 2 = moderate, 3 = strong and 4 = significantly strong.

### Statistical analysis

All experiments were performed in triplicate. Data were analyzed with SPSS 19.0 software (IBM, USA) and presented as the mean ± SEM. The statistical significance of the results was calculated using an unpaired Student’s t-test. DFS analysis was performed using the Kaplan-Meier method and log-rank test. Clinicopathological features were analyzed by a χ^2^ test. A Cox proportional hazards regression model was used to identify independent prognostic factors associated with DFS. Linear correlation analyzes were performed to determine correlations between circAKT3, miR-198 and PIK3R1 expression levels. A *P* value< 0.05 was defined as statistically significant.

## Results

### Ectopic circAKT3 expression levels are observed in CDDP-resistant GC cells and tissues and are correlated with poor prognosis in GC patients receiving CDDP therapy

**Fig. 1:**
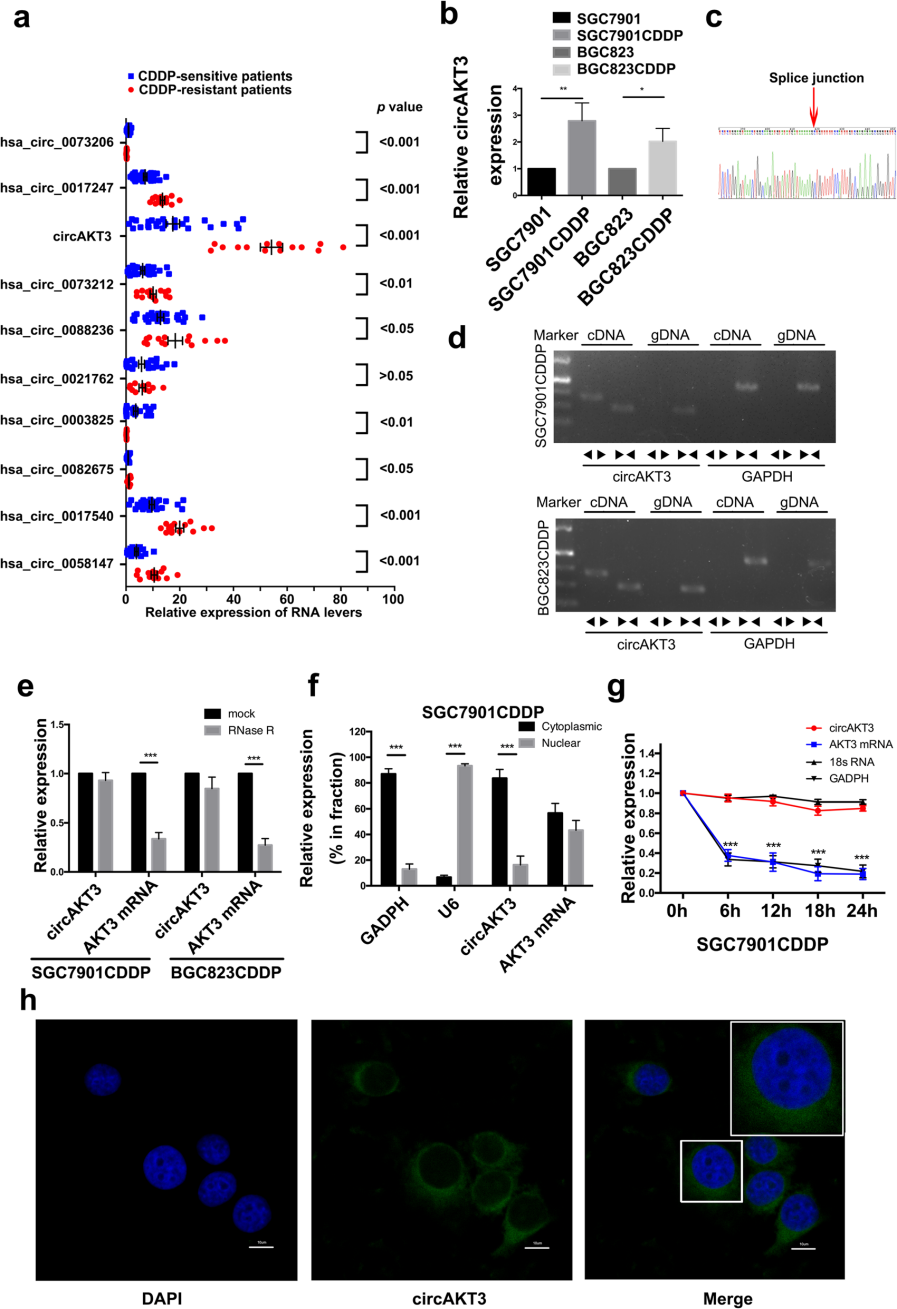
circAKT3 expression is increased in CDDP-resistant GC cells and tissues. **a** Validated expression of 10 circRNAs in the tissues from 44 GC patients using RT-qPCR. **b** Expression levels of circAKT3 in CDDP-resistant and their matched sensitive parental cell lines (SGC7901CDDP, BGC823CDDP, SGC7901 and BGC823) normalized to GAPDH expression. **c** The existence of circAKT3 was validated by Sanger sequencing. The red arrow shows the “head-to-tail” splicing sites of circAKT3. **d** The existence of circAKT3 was validated in SGC7901CDDP and BGC823CDDP cell lines by RT-PCR. Divergent primers amplified circAKT3 in cDNA but not in genomic DNA (gDNA). GAPDH served as a negative control. **e** RNA from SGC7901CDDP and BGC823CDDP cells was treated with or without RNase R for RT-qPCR. The relative levels of circAKT3 and AKT3 mRNA were normalized to the values measured in the mock-treated cells. **f** Levels of small nucleolar RNA (U6, as a positive control for the nuclear fraction), GAPDH (positive control for cytoplasmic fraction), AKT3 mRNA and circRNAs from the nuclear and cytoplasmic fractions of SGC7901CDDP cells. **g** RNA stability of circular and linear transcripts of AKT3 and of 18S rRNA in SGC7901CDDP cells. **h** Representative images of RNA FISH of circAKT3 expression in SGC7901CDDP cells, which show that circAKT3 is predominantly localized to the cytoplasm. Nuclei were stained with DAPI. Scale bar, 10 μm. The results are presented as the mean ± SEM. *P < 0.05, **P < 0.01, ***P < 0.001

To characterize circular RNA transcripts, we conducted RNA-Seq analysis of CDDP-resistant SGC7901 and BGC823 cells (i.e., SGC7901CDDP and BGC823CDDP) and their corresponding parental strains (i.e., SGC7901 and BGC823), which are sensitive to CDDP. The sequencing statistics are not shown. The analysis indicated that a series of circRNAs were differentially expressed in CDDP-resistant GC cells compared with the sensitive parental GC cells. We then chose the top 20 significantly upregulated circRNAs and verify their expression levels. Detailed information of 20 candidate circRNAs in Additional file 1: Table S8 (including location, genomic and spliced length). Using divergent primers to specifically target the circular junction as well as combined quantitative reverse transcription PCR (RT-qPCR) analysis and sequencing validation, we found that only 10 of these circRNAs had confirmed differences in expression and that circAKT3 was the most obviously upregulated circRNA in CDDP-resistant patients of cohort 1 (Fig. 1a and Additional file 2: Figure S1b-c). circAKT3 (hsa_circ_0000199) has been mapped to exons 8, 9, 10, and 11 of the AKT3 gene (555 bp) (Additional file 2: Figure S1d). Consistent with the RNA-Seq results, the expression of circAKT3 was obviously increased in CDDP-resistant GC cells (Fig. 1b). Subsequently, we verified the head-to-tail splicing of the RT-PCR product of circAKT3 by Sanger sequencing (Fig. 1c). Meanwhile, to exclude possibilities such as genomic rearrangements or trans-splicing, several experiments were employed. First, we designed convergent primers to amplify AKT3 mRNA and divergent primers to amplify circAKT3. Using cDNA and genomic DNA (gDNA) from SGC7901CDDP and BGC823CDDP cell lines as templates, the circAKT3 amplification product was only observed in cDNA by divergent primers but not in gDNA (Fig. 1d). In addition, the fragment of the linear form of AKT3 was digested by RNase R, but circAKT3 remained after RNase R treatment (Fig. 1e). Then, the relative expression levels of circAKT3 were detected in the cytoplasm and nucleus of SGC7901CDDP and BGC823CDDP cells (Fig. 1f and Additional file 2: Figure S1e). The RT-qPCR results demonstrated that circAKT3 was enriched in the cytoplasm. Moreover, we used Actinomycin D to suppress transcription and measure the half-life of circAKT3 in SGC7901CDDP and BGC823CDDP cells; we found that circAKT3 was more stable than AKT3 mRNA (Fig. 1g and Additional file 2: Figure S1f). Additionally, the FISH results displayed a dominantly cytoplasmic distribution of circAKT3 (Fig. 1h).

**Fig. 2:**
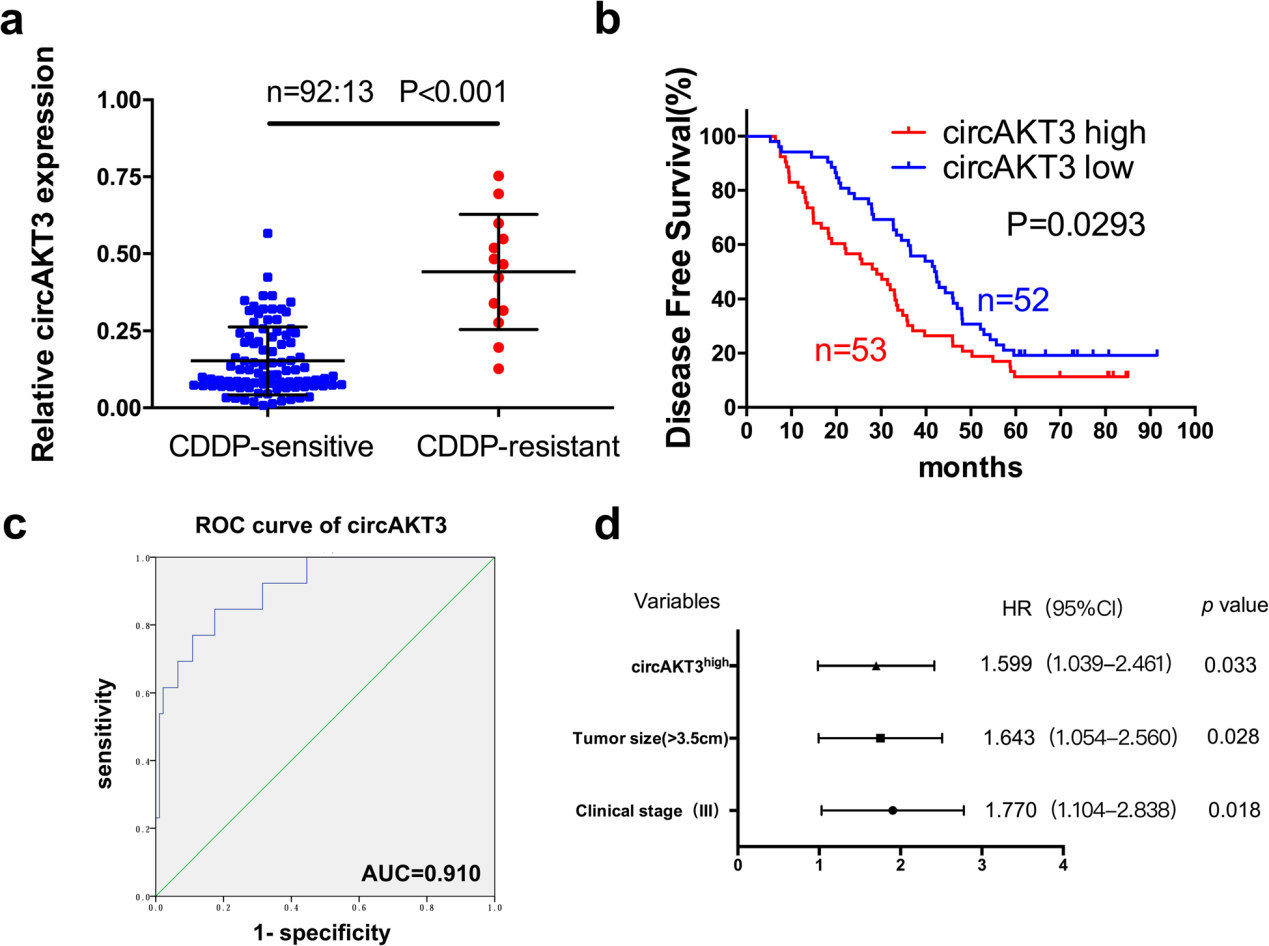
The circAKT3 expression level is correlated with poor prognosis in GC patients receiving CDDP therapy. **a** Expression levels of circAKT3 in tissue samples of 105 GC patients (CDDP-resistant and CDDP-sensitive groups) normalized to GAPDH expression. **b** Kaplan-Meier survival curves of DFS for patients with high (*n* = 53) or low (*n* = 52) circAKT3 expression. The median circAKT3 expression value was used as the cutoff. **c** ROC curves of circAKT3. **d** Multivariate analyses of hazard ratios for DFS. The results are presented as the mean ± SEM

Next, we detected the expression level of circAKT3 in tissues of patients from cohort 2. Consistent with the RNA-Seq results, circAKT3 was significantly more highly expressed in the CDDP-resistant GC tissues than in the sensitive tissues (Fig. 2a). Compared with GC patients expressing low levels of circAKT3, GC patients receiving CDDP therapy and exhibiting upregulation of circAKT3 showed a significant association with decreased five-year DFS (Fig. 2b). To further verify that circAKT3 may be a therapeutic target for CDDP-resistant patients, we calculated the area under the receiver operating characteristic curve (AUC) using the expression levels of circAKT3. The area under the curve is 91% (Fig. 2c), suggesting that the expression level of circAKT3 is a good predictive biomarker of CDDP resistance for GC patients. Analysis of the clinicopathological characteristics in cohort 2 showed that circAKT3 expression was positively related to tumor size, histological grade, clinical stage, T classification and CDDP resistance (Table 1). A univariate analysis showed that DFS was obviously related to tumor size, histological grade, clinical stage and circAKT3 expression level (Additional file 1: Table S9). Subsequently, multivariate analysis indicated that circAKT3 expression, along with tumor size and clinical stage, was an independent risk factor for DFS (Additional file 1: Table S9 and Fig. 2d).

**Table 1:** Correlation of relative circAKT3 expression with the clinicopathological characteristics of 105 patients accepted cisplatin-based chemotherapy with gastric cancer

Characteristics	Number	No. of patients		*P* value
		circAKT3^high^	circAKT3^low^	
Age(y)				
< 60	62	34	28	0.283
≥ 60	43	19	24	
Gender				
Male	80	37	43	0.121
Female	25	16	9	
Tumor size(cm)				
< 3.5	56	21	35	0.004**
≥ 3.5	49	32	17	
Histological grade				
Well-moderately	33	11	22	0.017*
Poorly-signet	72	42	30	
Clinical stage				
II	39	13	26	0.007**
III	66	40	26	
T classification				
T1-T2	31	9	22	0.004**
T3-T4	74	44	30	
N classification				
N0	14	5	9	0.232
N1-N3	91	48	43	
Cisplatin chemosensitivity				
Sensitive	92	41	51	0.001**
Resistant	13	12	1	

### circAKT3 facilitates CDDP resistance in vitro

**Fig. 3:**
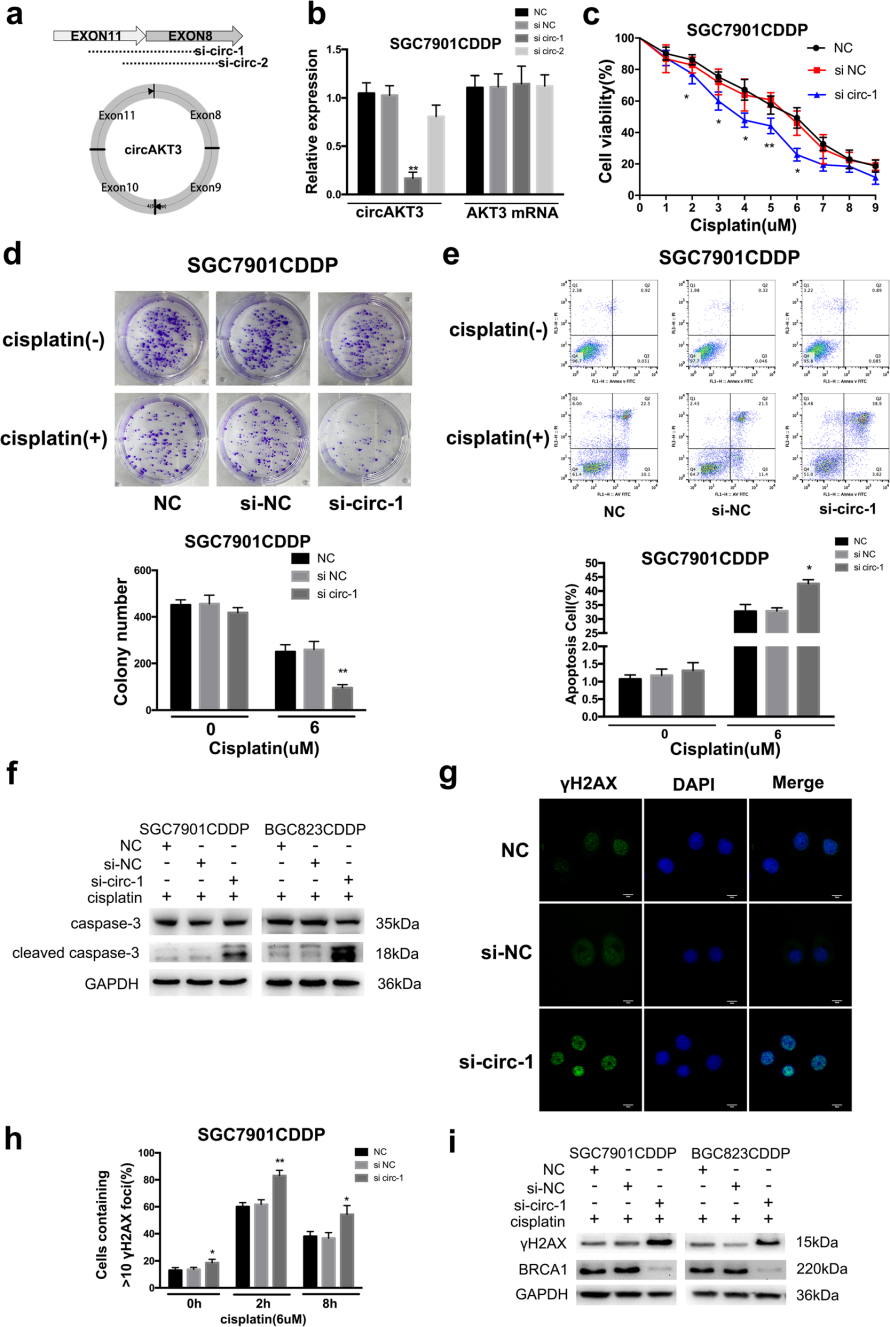
Downregulation of circAKT3 facilitates cisplatin sensitivity of CDDP-resistant GC cells in vitro*.* **a** Illustration showing the siRNA targeting the back-splice junction (si-circ-1 and si-circ-2). **b** RT-qPCR results for circular and linear transcripts of AKT3 in SGC7901CDDP cells treated with or without siRNA (NC, negative control; si-NC, control oligonucleotides with scramble sequence; si-circ-1 and si-circ-2, oligonucleotides targeting the back-splice junction). **c** Relative cell viability of NC, si-NC- transfected or si-circ-1-transfected SGC7901CDDP cells in the presence of CDDP at the indicated concentrations for 48 h. **d** Colony-forming ability of the NC, si-NC-transfected and si-circ-1-transfected SGC7901CDDP cells in the absence (Vehicle) or presence of CDDP (6 μM) for 48 h. **e** The apoptotic rates of NC SGC7901CDDP cells and SGC7901CDDP cells transfected with si-NC or si-circ-1 in the absence (Vehicle) or presence CDDP (6 μM) for 48 h were visualized by flow cytometry. **f** Western blot analysis shows apoptotic proteins in NC SGC7901CDDP cells and SGC7901CDDP cells transfected with si-NC or si-circ-1 upon CDDP treatment (6 μM) for 48 h (GAPDH was used as the loading control). **g** Immunofluorescence staining of γH2AX foci in NC SGC7901CDDP cells and SGC7901CDDP cells transfected with si-NC or si-circ-1 at 2 h after CDDP treatment (6 μM). Scale bars, 10 μm. **h** Percentage of cells containing > 10 γH2AX foci in NC SGC7901CDDP cells and SGC7901CDDP cells transfected with si-NC or si-circ-1 at 0 to 8 h after CDDP treatment (6 μM) removal. **i** Western blot analysis of γH2AX and BRCA1 expression in NC SGC7901CDDP and BGC823CDDP cells and cells transfected with si-NC or si-circ-1 after CDDP treatment (6 μM) removal. The results are presented as the mean ± SEM. *P < 0.05, **P < 0.01, ***P < 0.001

**Fig. 4:**
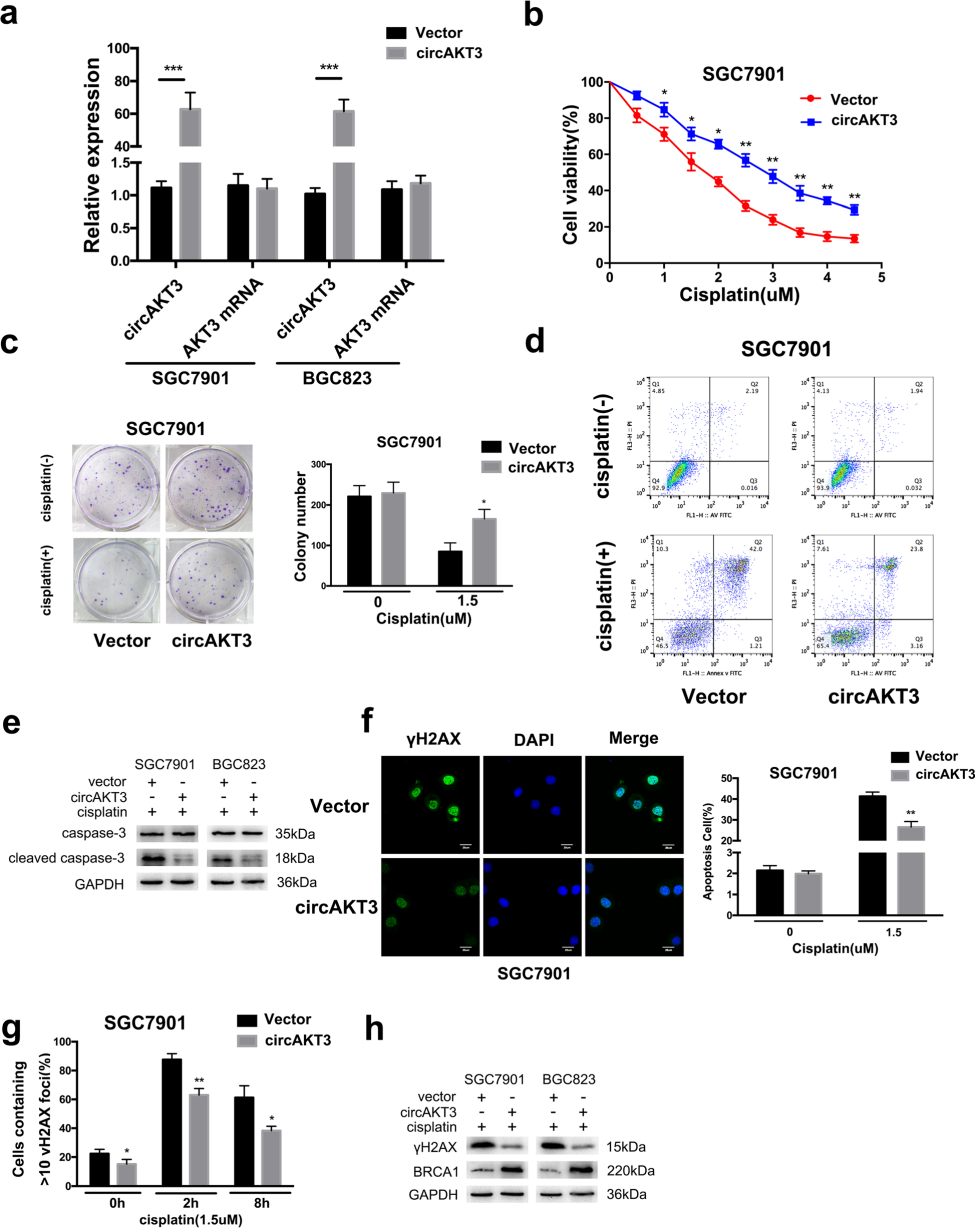
Overexpression of circAKT3 increases the resistance of CDDP-sensitive GC cells to cisplatin in vitro*.* **a** The expression levels of circAKT3 and AKT3 mRNA in SGC7901 and BGC823 cells after stable transfection of circAKT3 or empty vector plasmids were detected by RT-qPCR. **b** Relative cell viability of circAKT3 or vector-transfected SGC7901 cells exposed to CDDP at the indicated concentrations for 48 h. **c** Colony-forming ability of SGC7901 cells transfected with circAKT3 or vector in the absence (Vehicle) or presence of CDDP (1.5 μM) for 48 h. **d** The apoptosis rates of SGC7901 cells transfected with circAKT3 or vector after CDDP (1.5 μM) treatment for 48 h were detected by flow cytometry. **e** Western blot analysis of apoptotic proteins in SGC7901 cells transfected with circAKT3 or vector upon CDDP (1.5 μM) treatment for 48 h (GAPDH was used as the loading control). **f** Immunofluorescence staining of γH2AX foci in SGC7901 cells transfected with circAKT3 or vector at 2 h after CDDP treatment (1.5 μM). Scale bars, 10 μm. **g** Percentage of cells containing > 10 γH2AX foci in SGC7901 cells transfected with circAKT3 or vector at 0 to 8 h after CDDP (1.5 μM) treatment. **h** Western blot analysis of γH2AX and BRCA1 expression in SGC7901 cells transfected with circAKT3 or vector after CDDP treatment (1.5 μM) removal. The results are presented as the mean ± SEM. *P < 0.05, **P < 0.01, ***P < 0.001

First, we designed two siRNA oligonucleotides (si-circ-1 and si-circ-2) to target the unique back-splice junction of circAKT3 (Fig. 3a); si-circ-1 successfully knocked down circAKT3 expression but had no effect on the levels of endogenous linear AKT3 transcript in SGC7901CDDP and BGC823CDDP cells (Fig. 3b and Additional file 4: Figure S2a). Additionally, to further assess the role of circAKT3, circAKT3 was overexpressed in SGC7901 and BGC823 cells via transfection of pCD5-ciR-AKT3 e8–11 (Fig. 4a). Importantly, elevated expression of circAKT3 had no effect on the levels of linear AKT3 mRNA, as confirmed by RT-qPCR (Fig. 4a). circAKT3 inhibition reduced the viability of SGC7901CDDP and BGC823CDDP cells (Fig. 3c and Additional file 4: Figure S2b). In addition, knockdown of circAKT3 significantly decreased the number of cell colonies (Fig. 3d and Additional file 4: Figure S2c) and promoted apoptosis (Fig. 3e and Additional file 4: Figure S2d). The phosphorylated histone family member X (γH2AX) forms discrete nuclear foci and acts as a platform to recruit additional factors and enhance the DNA repair pathway [22]. Meanwhile, circAKT3-knockdown cells showed significantly more γH2AX foci than the control cells at 2 h after CDDP treatment (Fig. 3g and Additional file 4: Figure S2e). circAKT3-knockdown cells had a higher percentage of active foci relative to that in control cells from 0 to 8 h after CDDP treatment (Fig. 3h and Additional file 4: Figure S2f). However, compared with the negative control, ectopic circAKT3 expression significantly increased cell viability and the number of cell colonies and inhibited apoptosis and the formation of γH2AX foci in SGC7901 and BGC823 cells (Fig. 4b-g and Additional file 5: Figure S3a-e). We used western blotting to investigate the underlying mechanism of these activities. In the presence of CDDP, knockdown of circAKT3 in SGC7901CDDP and BGC823CDDP cells increased cleaved caspase-3 protein levels, while the levels of the inactivated form of caspase-3 protein was decreased (Fig. 3f). In contrast, cleaved and inactivated caspase-3 protein levels were observed when circAKT3 was overexpressed (Fig. 4e). These data are consistent with a previous study reporting that CDDP-induced increases in Breast cancer type 1 susceptibility protein(BRCA1) expression leads to enhanced DNA damage repair (DDR) in breast cancer cells [23]. After CDDP treatment, knockdown of circAKT3 in SGC7901CDDP and BGC823CDDP cells increased γH2AX but decreased BRCA1 protein levels. circAKT3 overexpression also inhibited γH2AX and promoted BRCA1 protein levels compared with the levels in the controls (Figs. 3i and 4h).

### circAKT3 exerts its function by sponging miR-198

**Fig. 5:**
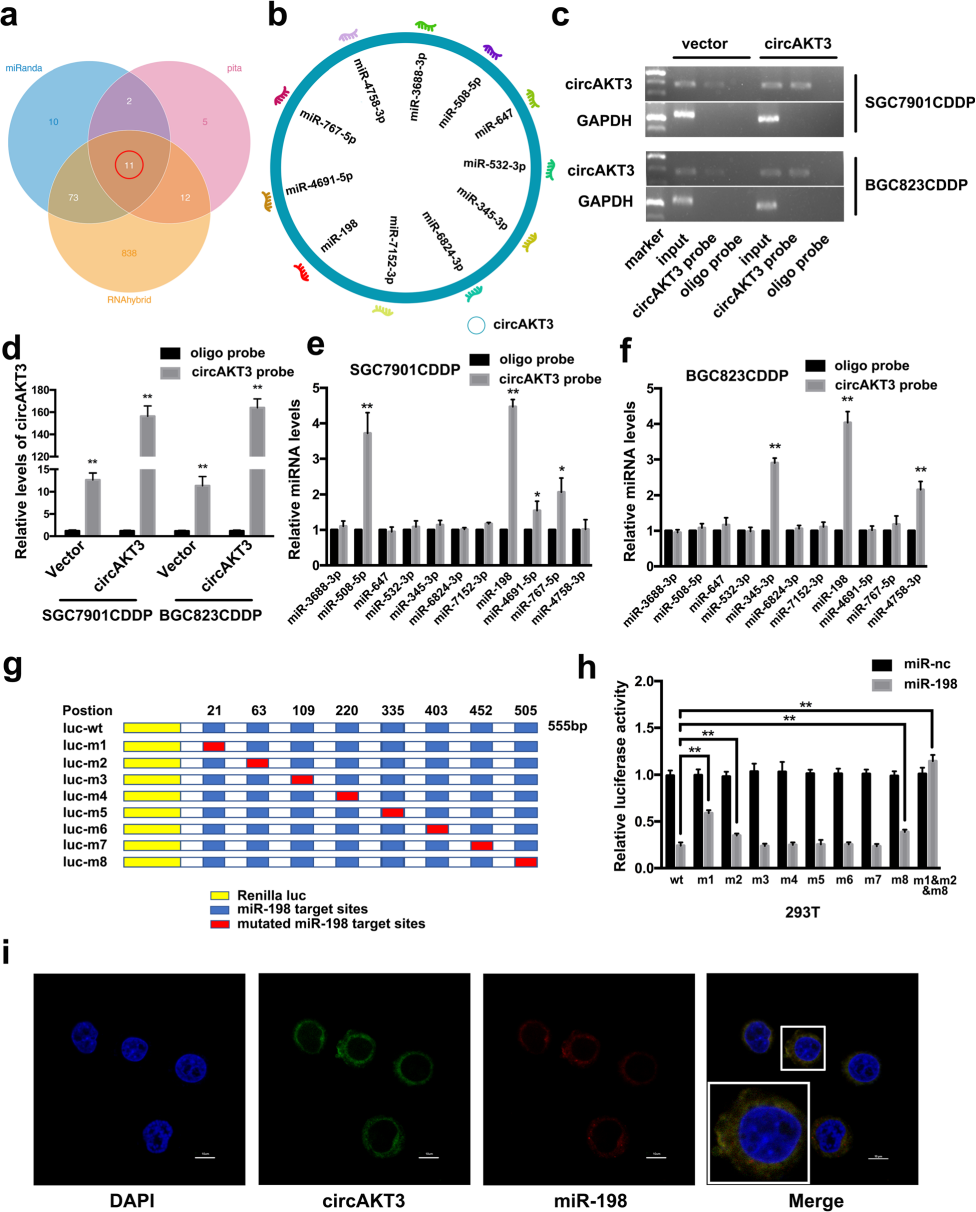
circAKT3 exerts its function by sponging miR-198. **a** & **b** Schematic illustration showing the overlap of the target miRNAs of circAKT3 predicted by miRanda, PITA and RNAhybrid. **c** & **d** Lysates prepared from SGC7901CDDP and BGC823CDDP cells stably transfected with circAKT3 or vector were subjected to RNA pull-down and tested by RT-PCR (C) and RT-qPCR (D). The relative level of circAKT3 was normalized to the input. GAPDH served as a negative control. **e** & **f** The relative levels of 11 miRNA candidates in SGC7901CDDP and BGC823CDDP lysates were detected by RT-qPCR. Multiple miRNAs were pulled down by circAKT3, and miR-198 was pulled down by circAKT3 in both cell lines. **g** Schematic illustration showing the 3′UTR of luciferase reporters containing the complete circAKT3 sequence (luc-wt) or the circAKT3 sequence with deletions of miR-198 (luc-m1-m8) binding sites. **h** Reporter assays showing the luciferase activity of luc-wt and luc-m1-m8 in 293 T cells cotransfected with miR-198 mimics or a scrambled oligonucleotide (control). **i** FISH showing the colocalization of circAKT3 and miR-198 in SGC7901CDDP cells. Nuclei were stained with DAPI. Scale bar, 10 μm. The results are presented as the mean ± SEM. *P < 0.05, **P < 0.01

To address whether circAKT3 could sponge miRNAs in GC cells, we selected 11 candidate miRNAs by overlapping the prediction results of the miRNA recognition elements in the circAKT3 sequence using miRanda, PITA, and RNAhybrid (Fig. 5a-b). Next, we investigated whether candidate miRNAs could directly bind circAKT3. A biotin-labeled circAKT3 probe was designed and verified to pull down circAKT3 in SGC7901CDDP and BGC823CDDP cell lines, and the pull-down efficiency was significantly enhanced in cells with stable circAKT3 overexpression (Fig. 5c-d). The miRNAs were extracted after pull-down, and the levels of the 11 candidate miRNAs were detected by RT-qPCR. As shown in Fig. 5e-f, in both SGC7901CDDP and BGC823CDDP cells, miR-198 was abundantly pulled down by circAKT3. Furthermore, using the RNAhybrid bioinformatics prediction tool, we calculated the secondary conformation of circAKT3 and miR-198 and found that there were 8 predicted binding domains (largest combined with a MFE > − 20 kcal/mol) (Additional file 3: Dataset S2). Next, the results of the luciferase reporter assays showed that miR-198 expression significantly reduced the luciferase activity of the reporter containing the complete circAKT3 sequence appended to the 3′-UTR of luciferase (luc-wt) compared to that of the reporter containing circAKT3 with mutated miR-198 binding sites (luc-m1, m2 and m8) (Fig. 5g-h). Moreover, RNA FISH assays revealed that circAKT3 and miR-198 were colocalized in the cytoplasm (Fig. 5i).

### PIK3R1 is a direct target of miR-198

**Fig. 6:**
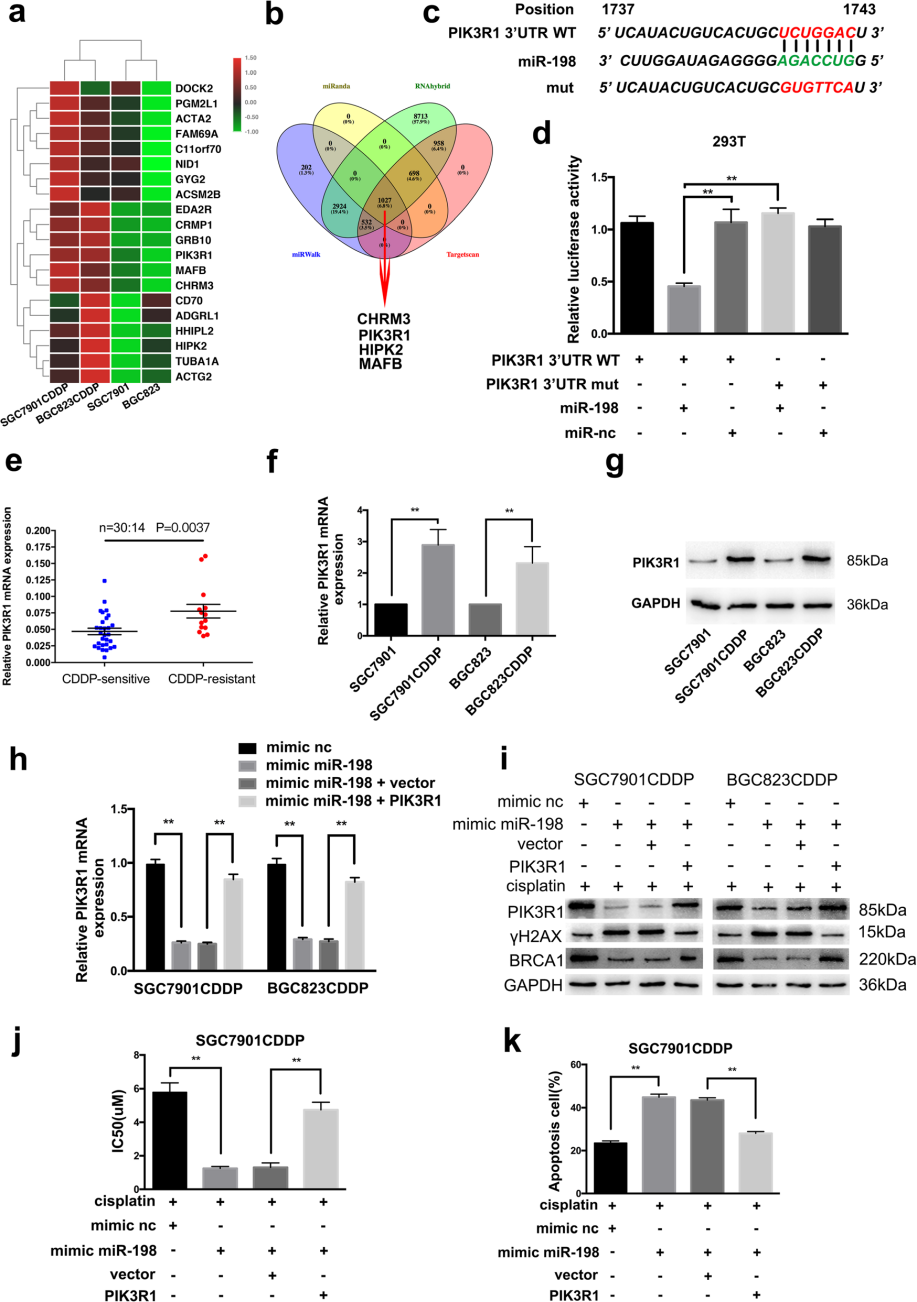
PIK3R1 is a direct target of miR-198. **a** mRNA microarray data of the top 20 upregulated genes in SGC7901CDDP, BGC823CDDP, SGC7901 and BGC823 cells are presented as heatmaps. **b** Venn diagram showing 4 genes that are putative miR-198 targets computationally predicted by four algorithms (miRanda, RNAhybrid, miRWalk and TargetScan) among the top 20 upregulated genes. **c** Schematic of PIK3R1 3’UTR wild-type (WT) and mutant (Mut) luciferase reporter vectors is shown. **d** The relative luciferase activities were analyzed in 293 T cells cotransfected with miR-198 mimics or miR-NC and luciferase reporter vectors PIK3R1 3’UTR (WT) or PIK3R1 3’UTR (Mut). **e** & **f** The expression of PIK3R1 was analyzed using RT-qPCR in tissues of cohort 1 (E) and in SGC7901CDDP, BGC823CDDP, SGC7901 and BGC823 cells (F). **g** The expression of PIK3R1 was analyzed using western blot in SGC7901CDDP, BGC823CDDP, SGC7901 and BGC823 cells. **h** & **i** The expression of PIK3R1 was analyzed by RT-qPCR (G) and western blot (H). SGC7901CDDP and BGC823CDDP cells were transfected with miR-198 mimic or cotransfected with the indicated vectors. **j** The IC50 of miR-198 was analyzed by the CCK8 assay. SGC7901CDDP cells were transfected with miR-198 mimic alone or cotransfected with the indicated vectors upon CDDP exposure (6 μM) for 48 h. **k** The apoptosis rates of SGC7901CDDP cells transfected with miR-198 mimic alone or cotransfected with the indicated vectors upon CDDP exposure (6 μM) for 48 h. The results are presented as the mean ± SEM. *P < 0.05, **P < 0.01

A microarray assay was further performed with SGC7901CDDP, BGC823CDDP, SGC7901 and BGC823 cells to validate the results of the ceRNA analysis. We analyzed the top 20 upregulated genes according to four algorithms (miRanda, RNAhybrid, miRWalk and TargetScan) prediction, and miR-198 could target the 3’UTRs of PIK3R1, CHRM3, HIPK2 and MAFB (Additional file 3: Dataset S3). We performed luciferase reporter assays to determine whether miR-198 directly targets these 4 genes in 293 T cells (Fig. 6c and Additional file 6: Figure S4c and e). In 293 T, SGC7901CDDP and BGC823CDDP cells cotransfected with miR-198 mimic, reporter constructs containing wild-type miR-198 binding sites at the PIK3R1 3′UTR exhibited decreased luciferase activity relative to that of reported constructs with mutated binding sites (Fig. 6d and Additional file 6: Figure S4f and g). PIK3R1 protein (p85α, encoded by PIK3R1) is the regulatory subunit of PI3K. A functional study demonstrated that PIK3R1 was highly expressed in CDDP-resistant ovarian cancer cells, and downregulated PIK3R1 resensitized the abovementioned cells to platinum-based treatment, which reveals the promising involvement of p85α in secondary CDDP resistance [24]. Compared with parental CDDP-sensitive cells, CDDP-resistant cells showed obvious increases in the expression of PIK3R1 mRNA and protein levels (Fig. 6f and g). Furthermore, we found that miR-198 mimics significantly inhibited PIK3R1 mRNA and protein levels and that ectopic PIK3R1 expression abolished the influence caused by miR-198 overexpression (Fig. 6h and i). Subsequently, the data showed that overexpression of miR-198 inhibited cell viability and induced apoptosis in SGC7901CDDP and BGC823CDDP cells. However, cotransfection of PIK3R1 and miR-198 abrogated these effects (Fig. 6j and k and Additional file 6: Figure S4h-j and Additional file 7: Figure S5a-b).

### circAKT3 regulates PIK3R1 expression, activates the PI3K/AKT signaling pathway and ultimately facilitates CDDP resistance by targeting miR-198 in vitro

**Fig. 7:**
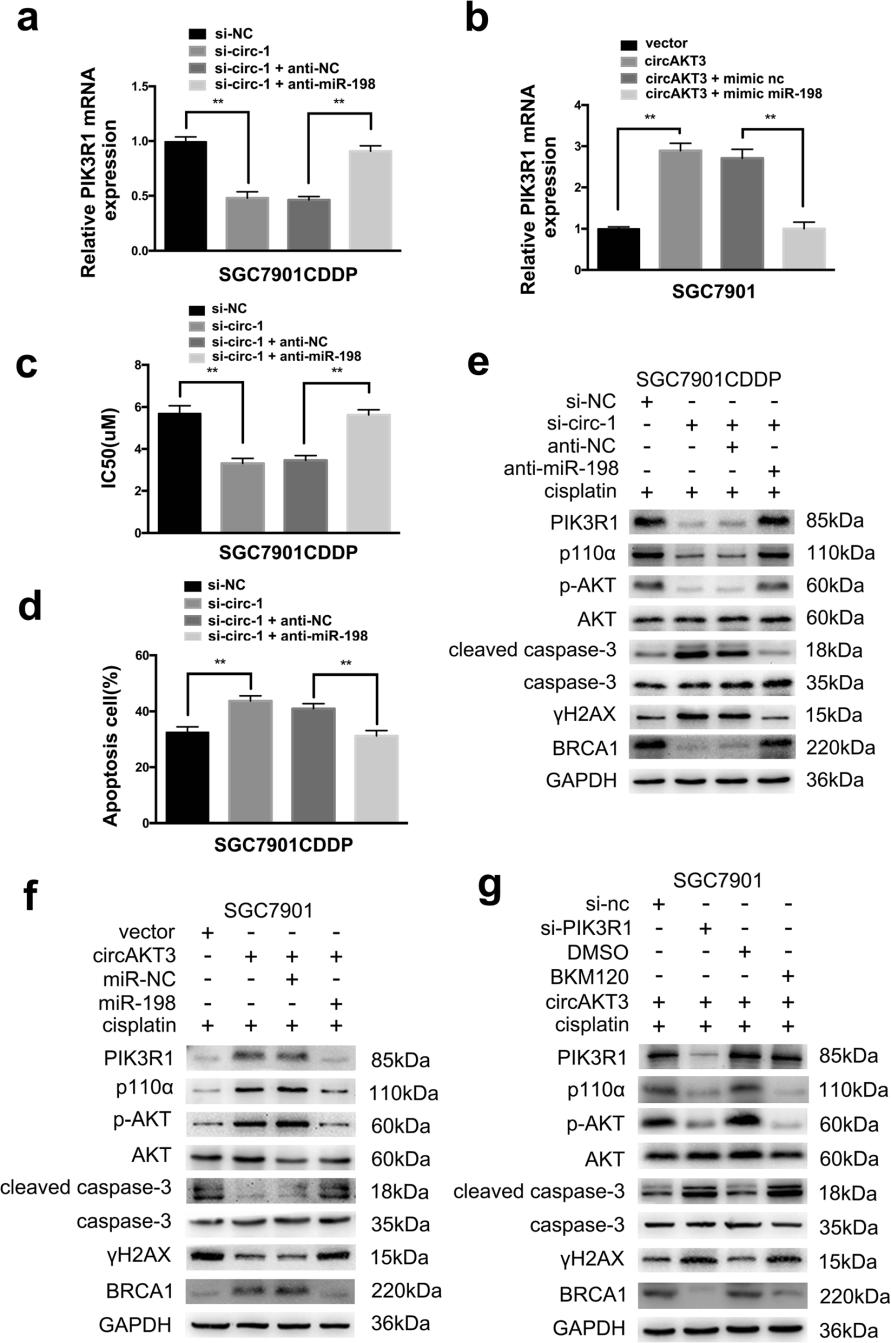
circAKT3 regulates PIK3R1 expression, induces cisplatin resistance and activates the PI3K/AKT signaling cascade by targeting miR-198 in vitro*.* **a** & **b** The expression levels of PIK3R1 were analyzed using RT-qPCR. SGC7901CDDP cells were transfected with indicated vectors alone or cotransfected the inhibitors, and SGC7901 cells were transfected with the indicated vectors and miR-198 mimics. **c** The IC50 of circAKT3 was analyzed by the CCK8 assay. SGC7901CDDP cells were transfected with inhibitor alone or cotransfected with the indicated vectors upon CDDP exposure (6 μM) for 48 h. **d** The apoptosis rates of SGC7901CDDP cells transfected with indicated vectors alone or cotransfected the inhibitors upon CDDP exposure (6 μM) for 48 h. **e** The expression levels of PIK3R1, apoptosis markers, γH2AX, BRCA1 and PI3K/AKT signaling molecules were determined using western blotting in SGC7901CDDP cells transfected with indicated vectors alone or cotransfected with the inhibitor after CDDP treatment (6 μM). **f** The expression levels of PIK3R1, apoptosis markers, γH2AX, BRCA1 and PI3K/AKT signaling molecules were determined using western blotting in SGC7901 cells transfected with the indicated vectors and miR-198 mimics after CDDP treatment (1.5 μM). Proteins were isolated from the indicated cells. **g** The expression levels of PIK3R1, apoptosis makers, γH2AX, BRCA1 and PI3K/AKT signaling molecules were determined using western blotting in SGC7901 cells transfected with the indicated vectors and BKM20 after CDDP treatment (1.5 μM). The results are presented as the mean ± SEM. *P < 0.05, **P < 0.01

Cotransfection of si-circ-1 and anti-miR-198 could counteract the si-circ-1-induced downregulation of PIK3R1 in SGC7901CDDP cells (Fig. 7a). Notably, cotransfection of circAKT3 and miR-198 attenuated the expression of PIK3R1 compared to transfection of circAKT3 alone in SGC7901 cells (Fig. 7b). The CCK8 and flow cytometry analyses indicated that transfection with si-circ-1 inhibited cell viability and promoted apoptosis after CDDP treatment while cotransfection with si-circ-1 and anti-miR-198 significantly promoted cell viability and inhibited apoptosis compared with the controls (Fig. 7c and d, Additional file 7: Figure S5d). Furthermore, upregulation of circAKT3 led to enhanced cell viability, but this effect could be significantly abolished by ectopic expression of miR-198 (Additional file 8: Figure S6a). Additionally, overexpression of circAKT3 induced a reduction in apoptosis. However, cotransfection of circAKT3 and miR-198 mimics led to enhanced apoptosis (Additional file 8: Figure S6b). Notably, circAKT3 upregulation could inhibit γH2AX expression, as indicated by the reduced fluorescence of γH2AX, cotransfection of circAKT3 and miR-198 mimics led to abolish this effect (Additional file 8: Figure S6c and d). Transfection of si-circ-1 significantly reduced PIK3R1 expression and inhibited the expression of canonical PI3K/AKT signaling molecules, as shown by western blot, and downregulating of both circAKT3 and miR-198 abrogated these effects in SGC7901CDDP cells (Fig. 7e). Similar results are presented in Additional file 9: Figure S7a-f. Meanwhile, Transfection of circAKT3 significantly increased PIK3R1 expression and induced the expression of canonical PI3K/AKT signaling molecules, as shown by western blot, and concomitant overexpression of both circAKT3 and miR-198 abrogated these effects in SGC7901 cells (Fig. 7f). Transfection of si-PIK3R1 significantly inhibited PIK3R1 and levels of phosphorylated PI3K/AKT signaling molecules in SGC7901 cells with circAKT3 overexpression. We employed a specific p110α inhibitor, BKM20, and performed western blotting to determine whether deactivation of PI3K/AKT signaling can overcome the changes caused by circAKT3 overexpression. The results indicated that si-PIK3R1 and BKM20 significantly inhibited PIK3R1 and p110α levels, respectively as well as inhibited p-AKT, reduced BRCA1 levels, increased cleaved caspase-3 levels, and promoted γH2AX (Fig. 7g). These results suggest that circAKT3 functions by targeting miR-198 as a ceRNA to regulate PIK3R1 expression, activate the PI3K/AKT signaling cascade and facilitate CDDP resistance.

### circAKT3 promotes CDDP resistance of GC cells in vivo

**Fig. 8:**
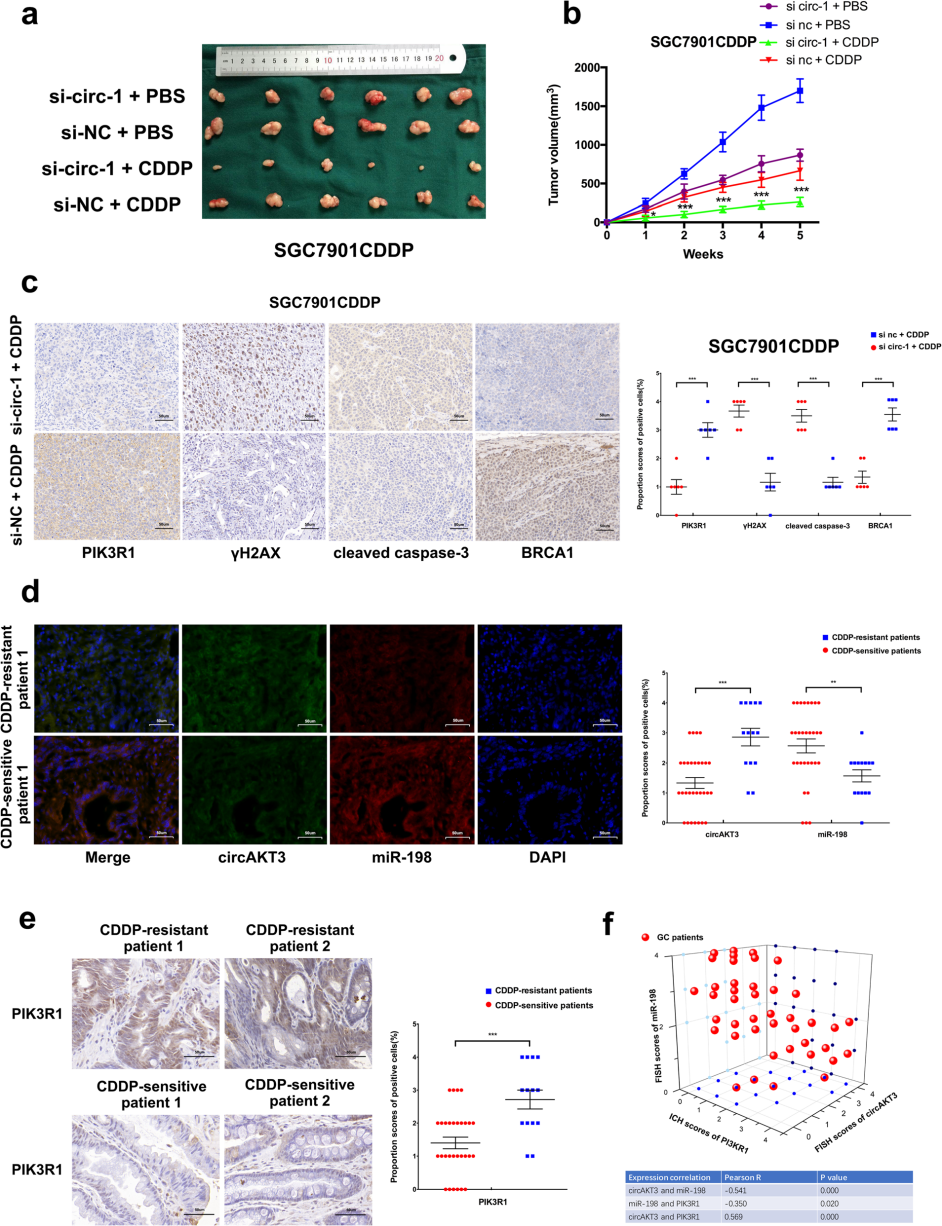
circAKT3 promotes cisplatin resistance of GC cells in vivo. **a** Xenograft tumors of sacrificed mice with or without CDDP treatment (3 mg/kg, three times a week) at the end of the experiment. **b** Growth curves of subcutaneous xenograft tumors. **c** PIK3R1, γH2AX, cleaved caspase-3 and BRCA1 expression levels are shown in representative xenograft tumors by IHC (Left) (400x magnification, scale bars = 50 μm). Quantification of the IHC scores of PIK3R1, γH2AX, cleaved caspase-3 and BRCA1 expression levels (Right). **d** FISH showing the colocalization of circAKT3 and miR-198 in CDDP-resistant or CDDP-sensitive GC tissues from patients. FISH scores of circAKT3 and miR-198 were further calculated in 14 CDDP-resistant and 30 CDDP-sensitive patient tissues. Nuclei were stained with DAPI. Scale bar, 10 μm. **e** IHC staining of PIK3R1 in CDDP-resistant or CDDP-sensitive GC tissues from patients (400x magnification, scale bars = 50 μm). The IHC scores of PIK3R1 were further determined in 14 CDDP-resistant and 30 CDDP-sensitive patient tissues. **f** Three-dimensional scatter plot of circAKT3, miR-198 and PIK3R1 levels in 14 CDDP-resistant and 30 CDDP-sensitive GC tissues from patients. The results are presented as the mean ± SEM. *P < 0.05, **P < 0.01, ***P < 0.001

To investigate the potential clinical relevance of circAKT3 in vivo, we subcutaneously injected SGC7901CDDP cells with or without stable circAKT3 knockdown (Additional file 8: Figure S6e) into the dorsal flanks of female BALB/c nude mice and allowed the cells to proliferate for 5 weeks. Tumor xenograft data indicated that circAKT3 inhibition in CDDP-resistant cells can significantly decrease xenograft tumor growth and sensitize cells to CDDP treatment (Fig. 8a and b). IHC analysis of tumor xenograft samples further indicated that the protein levels of γH2AX and cleaved caspase-3 were notably increased, but BRCA1 was decreased upon circAKT3 inhibition (Fig. 8c). FISH showed the colocalization of circAKT3 and miR-198 in tissues from patients with CDDP-resistant or CDDP-sensitive GC (Fig. 8d). The FISH score data showed that the expression of circAKT3 was significantly higher in CDDP-resistant GC tissues than CDDP-sensitive GC tissues; however, miR-198 expression showed the opposite result (Fig. 8d). Similarly, IHC scores and western blot analyses indicated that PIK3R1 protein expression was obviously increased in CDDP-resistant GC tissues compared to CDDP-sensitive GC tissues (Fig. 8e and Additional file 10: Figure S8a and b). Furthermore, correlations were identified between circAKT3 and miR-198 expression levels and PIK3R1 protein levels in these 44 GC tissue samples (Fig. 8f). Based on these data, we conclude that circAKT3 increases the tolerance of GC cells to CDDP by targeting PIK3R1 through miR-198.

## Discussion

CDDP treatment is one of the most predominant chemotherapeutic strategies for patients with GC [25]. In this study, using RNA-Seq analysis, we determined that circRNA expression is associated with CDDP resistance in GC. We found a novel circular RNA termed circAKT3 that was upregulated in tissue samples from patients with CDDP-resistant GC and in CDDP-resistant cell lines and was correlated with five-year DFS. Moreover, circAKT3 was expressed at higher levels than other candidate circRNAs in CDDP-resistant GC patients, which meant that it may play a more important role than other circRNAs in GC.

DNA is the recognized target for CDDP cytotoxicity in cancer therapy. The resultant biological process in response to CDDP and other DNA-damaging therapies is the activation of apoptosis and the destruction of malignant cells. The most favorable evidence is the hypersensitivity of both eukaryotic and prokaryotic cells deficient in DNA repair to CDDP [26]. An enhanced DNA repair mechanism can induce the survival of damaged or mutated tumor cells, resulting in resistance and subsequent tumor recurrence [27]. BRCA1, a tumor-suppressor gene, is widely involved in cellular metabolism [28], transcriptional regulation [29, 30], and epigenetic modification [31]. A growing body of evidence revealed that BRCA1 has a great effect on the modulation of CDDP resistance [32–34]. Alterative BRCA1 expression can regulate the mitochondrial fission program, which could modulate CDDP sensitivity [32]. Some microRNAs contribute to DNA repair and CDDP sensitivity through BRCA1 deregulation, including miR-9 [35] and miR-638 [36]. A previous study demonstrated that BRCA1 mRNA levels were negatively associated with CDDP sensitivity in GC [37]. Currently, there is a lack of research on circRNAs that regulate the BRCA1 gene in tumor cells, including GC. The underlying mechanisms require further exploration.

PIK3R1 encodes the regulatory subunit of PI3K (p85α). Some studies have reported that p85α positively regulates PI3K signaling in CDDP resistance. Because the PI3K pathway is a critical player in tumorigenesis and the ubiquitously hyperactivated signaling pathway in neoplasms, its inhibition both pharmacologically and genetically is considered to be the most promising strategy for targeted cancer treatment [38]. The PI3K pathway has been revealed as a mediator of platinum resistance [39, 40] For instance, AKT activation mediated resistance to caspase-independent CDDP-induced apoptosis through inhibiting the apoptosis-inducing factor-associated pathway [40]. One study reported that PI3K/AKT activation induced the upregulation of BRCA1 in tamoxifen-resistant breast cancer cells and resensitized them to CDDP treatment [23]. Nevertheless, the underlying mechanism of how PIK3R1 mediates resistance to chemotherapy and whether this involves canonical PI3K signaling and downstream BRCA1 activity remains to be investigated in GC.

Based on the abovementioned studies, we conducted a series of experiments and demonstrated that circAKT3 reduced CDDP-induced activation of caspase-3 and apoptosis, leading to enhanced DDR and resistance to CDDP chemotherapy. Mechanistically, circAKT3 functions as a ceRNA by sponging miR-198 to abolish the suppressive effect of this miRNA on its target gene PIK3R1, which activated the PI3K/AKT signaling pathway in GC cells. The study showed that PI3K/AKT pathway activation contributes to upregulation of the DNA repair molecule BRCA1 and leads to resistance to CDDP-based DNA-damaging chemotherapy [23]. In the current study, circAKT3 influenced DDR in GC cells, implying that circAKT3 might enhance CDDP resistance through the PI3K/AKT pathway and DDR mechanisms in GC cells. However, there are some drawbacks in this study, and we have not yet identified the specific mechanism by which BRCA1 regulates DDR in GC. At the same time, the reasons for circAKT3 shear formation and the upstream regulatory mechanism were not discussed. For the in vivo experiments, we did not use animal models of GC in situ, nor did we fully simulate the process of drug resistance. These approaches should be further pursued in subsequent studies.

## Conclusions

In conclusion, we show that circAKT3 is upregulated in human GC and that it can efficiently sponge miR-198 to restore PIK3R1 expression. We also demonstrate that downregulation of circAKT3 can effectively promote CDDP sensitivity in GC cells by targeting the miR-198/PIK3R1 axis. Our results provide novel evidence that circRNAs function as “microRNA sponges” and highlight a promising therapeutic target for the CDDP resistance of GC patients.

## Additional files

Additional file 1: **Table S1.** Detailed information of cohort 1 and 2 is listed. **Table S2.** Primers and RNA sequences used in this study. **Table S3.** SiRNA sequences used in this study. **Table S4.** MiRNA mimics, and inhibitors sequences used in this study. **Table S5.** Biotinylated probes sequences used in this study. **Table S6.** Fish probes sequences used in this study. **Table S7.** Detailed information of antibody used in this study. **Table S8.** Detailed information of 20 candidate circRNAs (including location, genomic and spliced length). **Table S9.** Univariate and multivariate analysis of cohort 2. (DOCX 31 kb)

Additional file 2: **Figure S1. a** SGC7901CDDP/SGC7901 and BGC823CDDP/BGC823 cell viability in response to different concentrations of cisplatin. **b** RT-PCR products with divergent primers showing a single, distinct product of the expected size. **c** Melting curves of RT-qPCR product of verified circRNAs, indicating the specificity of RT-qPCR products with no primer dimers or nonspecific amplified products. **d** Schematic illustrating that circAKT3 (hsa_circ_0000199) is derived from exons 8, 9, 10, and 11 of the AKT3 gene (555 bp). **e** Levels of small nucleolar RNA (U6, as a positive control for the nuclear fraction), GAPDH (positive control for the cytoplasmic fraction), AKT3 mRNA and circRNAs from nuclear and cytoplasmic fractions of BGC823CDDP cells. **f** RNA stability of the circular and linear transcripts of AKT3 and 18S rRNA in BGC823CDDP cells. The results are presented as the mean ± SEM. *P<0.05, **P<0.01, ***P<0.001. (TIF 2497 kb)

Additional file 3: **Additional Dataset (1)** miRNA target prediction of circAKT3. **Additional Dataset (2)** Eight miR-198 binding sites mutation of circAKT3. **Additional Dataset (3)** mRNA target prediction of miR-198. (ZIP 1800 kb)

Additional file 4: **Figure S2. a** RT-qPCR results for the circular and linear transcripts of AKT3 in BGC823CDDP cells treated with or without siRNA (NC, negative control; si-NC, control oligonucleotides with scramble sequence; si-circ-1 and si-circ-2, oligonucleotides targeting the back-splice junction). **b** Relative cell viability of NC BGC823CDDP cells and BGC823CDDP cells transfected with si-NC- or si-circ-1 after CDDP treatment at the indicated concentrations for 48 h. **c** Colony-forming ability of the NC BGC823CDDP cells and si-NC- or si-circ-1-transfected BGC823CDDP cells in the absence (Vehicle) or presence of CDDP (15 μM) for 48 h. **d** The apoptosis rates of NC BGC823CDDP cells and BGC823CDDP cells transfected with si-NC or si-circ-1 in the absence (Vehicle) or upon CDDP (15 μM) for 48 h by flow cytometry. **e** Immunofluorescence staining of γH2AX foci in NC BGC823CDDP cells and BGC823CDDP cells transfected with si-NC or si-circ-1 at 2 h after CDDP treatment (15 μM). Scale bars, 10 μm. **f** Percentage of cells containing >10 γH2AX foci in NC BGC823CDDP cells and BGC823CDDP cells transfected with si-NC or si-circ-1 at 0 to 8 h after CDDP treatment (15 μM) removal. The results are presented as the mean ± SEM. *P<0.05, **P<0.01, ***P<0.001. (TIF 5287 kb)

Additional file 5: **Figure S3. a** Relative cell viability of circAKT3- or vector-transfected BGC823 cells with CDDP treatment at the indicated concentrations for 48 h. **b** Colony-forming ability of BGC823 cells transfected with circAKT3 or vector in the absence (Vehicle) or presence of CDDP (1.5 μM) for 48 h. **c** The apoptosis rates of BGC823 cells transfected with circAKT3 or vector upon CDDP (1.5 μM) for 48 h by flow cytometry. **d** Immunofluorescence staining of γH2AX foci in BGC823 cells transfected with circAKT3 or vector at 2 h after CDDP treatment (1.5 μM). Scale bars, 10 μm. **e** Percentage of cells containing >10 γH2AX foci in BGC823 cells transfected with circAKT3 or vector at 0 to 8 h after CDDP treatment (1.5 μM) removal. The results are presented as the mean ± SEM. *P<0.05, **P<0.01, ***P<0.001. (TIF 3594 kb)

Additional file 6: **Figure S4. a** Predicted secondary structure of circAKT3 using the Vienna RNA package. **b** The expression of miR-198 was analyzed using RT-qPCR in tissues of cohort 1. **c**, **d, e** The relative luciferase activities were analyzed in 293T cells cotransfected with miR-198 mimics or miR-NC and luciferase reporter vectors containing the WT or Mut 3’UTR of CHRM3 (C), HIPK2 (D), and MAFB (E). **f** & **g** The relative luciferase activities were analyzed in SGC7901CDDP(**f**) and BGC823CDDP(**g**) cells cotransfected with miR-198 mimics or miR-NC and luciferase reporter vectors PIK3R1 3’UTR (WT) or PIK3R1 3’UTR (Mut). **h** & **i** The expression levels of PIK3R1 (**h**) and miR-198 (**i**) in SGC7901CDDP and BGC823CDDP cells after transfection of PIK3R1 plasmids or miR-198 mimics were detected by RT-qPCR. **j** The IC50 of miR-198 was analyzed by the CCK8 assay. BGC823CDDP cells were transfected with miR-198 mimic alone or cotransfected with the indicated vectors upon CDDP exposure (15 μM) for 48 h. The results are presented as the mean ± SEM. *P<0.05, **P<0.01, ***P<0.001. (TIF 1650 kb)

Additional file 7: **Figure S5. a** Apoptotic flow cytometry. SGC7901CDDP cells were transfected with miR-198 mimic alone or cotransfected with the indicated vectors upon CDDP exposure (6 μM) for 48 h **b** The apoptosis rate was analyzed by flow cytometry. BGC823CDDP cells were transfected with miR-198 mimic alone or cotransfected with the indicated vectors upon CDDP exposure (15 μM) for 48 h. **c** The expression levels of miR-198 in SGC7901CDDP and BGC823CDDP cells after transfection of anti-miR-198 were detected by RT-qPCR. **d** Apoptotic flow cytometry. SGC7901CDDP cells transfected with indicated vectors alone or cotransfected the inhibitors upon CDDP exposure (6 μM) for 48 h. The results are presented as the mean ± SEM. *P<0.05, **P<0.01, ***P<0.001. (TIF 2595 kb)

Additional file 8: **Figure S6. a** The IC50 was analyzed by CCK8 assay, SGC7901 cells were transfected with inhibitor alone or cotransfected with the indicated vectors upon CDDP exposure (1.5 μM) for 48 h. **b** The apoptosis rate was analyzed by flow cytometry. SGC7901 cells were transfected with inhibitor alone or cotransfected with the indicated vectors upon CDDP exposure (1.5 μM) for 48 h. **c** Immunofluorescence staining of γH2AX foci in SGC7901 cells transfected with inhibitor alone or cotransfected with the indicated vectors at 2 h after CDDP treatment (1.5 μM). Scale bars =10 μm. **d** Percentage of cells containing >10 γH2AX foci in SGC7901 cells transfected with inhibitor alone or cotransfected with the indicated vectors at 0 to 8 after CDDP treatment (1.5 μM) removal. **e** SGC7901CDDP cells stably expressing circAKT3 siRNA (si-circ-1) and its negative control siRNA (si-NC) were generated by infection with lentiviruses, the expression levels of cricAKT3 were detected by RT-qPCR. The results are presented as the mean ± SEM. *P<0.05, **P<0.01, ***P<0.001. (TIF 2160 kb)

Additional file 9: **Figure S7. a** The expression levels of PIK3R1 were analyzed using RT-qPCR. SGC7901CDDP cells were cotransfected with inhibitors alone or the indicated vectors. **b** The IC50 of circAKT3 was analyzed by the CCK8 assay. SGC7901CDDP cells were transfected with inhibitor alone or cotransfected with the indicated vectors upon CDDP exposure (6 μM) for 48 h. **c** The apoptosis rates of SGC7901CDDP cells transfected with inhibitor alone or cotransfected with the indicated vectors upon CDDP exposure (6 μM) for 48 h. **d** Immunofluorescence staining of γH2AX foci in SGC7901CDDP cells transfected with inhibitor alone or cotransfected with the indicated vectors at 2 h after CDDP treatment (6 μM). Scale bars, 10 μm. **e** Percentage of cells containing >10 γH2AX foci in SGC7901CDDP cells transfected with inhibitor alone or cotransfected with the indicated vectors at 0 to 8 after CDDP treatment (6 μM) removal. **f** The expression levels of PIK3R1, apoptosis markers, γH2AX, BRCA1 and PI3K/AKT signaling molecules were determined using western blotting in SGC7901CDDP cells transfected with inhibitor alone or cotransfected with the indicated vectors after CDDP treatment (6 μM). The results are presented as the mean ± SEM. *P<0.05, **P<0.01, ***P<0.001. (TIF 2258 kb)

Additional file 10: **Figure S8.** a The expression of PIK3R1 were analyzed using western blot in tissue of cohort 1. b The expression of PIK3R1 were further determined in tissues of 14 CDDP-resistant and 30 CDDP-sensitive patients (Relative to GAPDH). (TIF 604 kb)

## Abbreviations

AUC: Area under the receiver operating characteristic curve
BRCA1: Breast cancer type 1 susceptibility protein
CCK8: Cell Counting Kit-8
CDDP: Cisplatin
ceRNAs: Competitive endogenous RNAs
circRNAs: Circular RNAs
DDR: DNA damage repair
DFS: Disease-free survival
FBS: Fetal bovine serum
FISH: Fluorescence in situ hybridization
FL: Firefly luciferase
GC: Gastric cancer
gDNA: Genomic DNA
IHC: Immunohistochemical staining
MFE: Minimum free energy
miRNAs: MicroRNAs
mtDNA: Mitochondrial DNA
ncRNAs: Noncoding RNAs
RNA-Seq: RNA sequencing
RT-PCR: Quantitative real-time PCR
RT-qPCR: Quantitative reverse transcription PCR
γH2AX: Phosphorylated histone family member X
