# Supplementary material for: Circular RNA AKT3 upregulates PIK3R1 to enhance cisplatin resistance in gastric cancer via miR-198 suppression
Source: Mol Cancer. 2019 Mar 30;18:71. doi: 10.1186/s12943-019-0969-3 (PMC6441201; doi:10.1186/s12943-019-0969-3)
Supplement: Supplementary file 1 — Table S1. Detailed information of cohort 1 and 2 is listed. Table S2. Primers and RNA sequences used in this study. Table S3. SiRNA sequences used in this study. Table S4. MiRNA mimics, and inhibitors sequences used in this study. Table S5. Biotinylated probes sequences used in this study. Table S6. Fish probes sequences used in this study. Table S7. Detailed information of antibody used in this study. Table S8. Detailed information of 20 candidate circRNAs (including location, genomic and spliced length). Table S9. Univariate and multivariate analysis of cohort 2. (DOCX 31 kb) [file 12943_2019_969_MOESM1_ESM.docx]

**Additional file 1 legends,**

**Table S1,** Detailed information of cohort 1 and 2 is listed.

**Table S2,** Primers and RNA sequences used in this study.

**Table S3,** SiRNA sequences used in this study.

**Table S4,** MiRNA mimics, and inhibitors sequences used in this study.

**Table S5,** Biotinylated probes sequences used in this study.

**Table S6,** FISH probes sequences used in this study.

**Table S7,** Detailed information of antibody used in this study.

**Table S8,** Detailed information of 20 candidate circRNAs (including location, genomic and spliced length).

**Table S9,** Univariate and multivariate analysis of cohort 2.

**Table S1**

|  | Cohort1 | Cohort2 |
| --- | --- | --- |
| All cases | 44 | 105 |
| Age(y), ≥ 60: < 60 | 28:16 | 43:62 |
| Gender, Male: Female | 37:7 | 80:25 |
| Tumor size(cm), ≥ 3.5: < 3.5 | 21:23 | 49:56 |
| Histological grade, Well-moderately: Poorly-signet | 15:29 | 33:72 |
| Clinical stage, II: III | 14:30 | 39:66 |
| T classification, T1-T2: T3-T4 | 15:29 | 31:74 |
| N classification, N0: N1-N3 | 9:35 | 14:91 |
| Cisplatin chemosensitivity, Sensitive: Resistant | 30:14 | 92:13 |

**Table S2**

| **Primer sequence** |  |  |
| --- | --- | --- |
| hsa_circ_0073206 | Forward | GGTCCCATGGGTGGATTAGG |
| hsa_circ_0073206 | Reverse | AGAGTGCTAACCTTGGAAATACTGT |
| hsa_circ_0017247 | Forward | GAAATTGTCTCTGCCTTGGACT |
| hsa_circ_0017247 | Reverse | TCCTATGAATGAGCCATCTGTCT |
| hsa_circ_0073212 | Forward | TGCCCTTAGGACCACAGAA |
| hsa_circ_0073212 | Reverse | GAGATCCCAAAATACATGAATTG |
| hsa_circ_0088236 | Forward | TCCAGATACGGCGGGATGA |
| hsa_circ_0088236 | Reverse | CGTGTAGACACCACAGTCCTT |
| hsa_circ_0021762 | Forward | CTGTGAAGTGAAGCCAAAGCC |
| hsa_circ_0021762 | Reverse | TTTGGACACATGATCCTCTCTTCC |
| hsa_circ_0003825 | Forward | TTGAAATCACACCAACTTCCTCTCG |
| hsa_circ_0003825 | Reverse | AGGAATGTCAACACTTCAAACCATGT |
| hsa_circ_0082675 | Forward | CCCGTTCCAGCAAGCTCTCA |
| hsa_circ_0082675 | Reverse | CTGGAAACATGATTTGACGCTTGG |
| hsa_circ_0017540 | Forward | AAAAAGCACAAGCGAACCCCA |
| hsa_circ_0017540 | Reverse | GAATTGCTCCAAAGCTGCACG |
| hsa_circ_0058147 | Forward | GGAGAAGTATGTGCATGGTGTCA |
| hsa_circ_0058147 | Reverse | TGCAGATTTCCTCGTGGGTTG |
| circAKT3 | Forward | TCCAAATAAACGCCTTGGTGG |
| circAKT3 | Reverse | CCTCAGAGAACACCCGCTCT |
| AKT3 | Forward | GGAGTCATCATGAGCGATGTT |
| AKT3 | Reverse | AAGGAAGTATCTTGGCCTCCA |
| GAPDH | Forward | CAATGACCCCTTCATTGACC |
| GAPDH | Reverse | TTGATTTTGGAGGGATCTCG |
| Divergent GAPDH | Forward | GAAGGTGAAGGTCGAGTC |
| Divergent GAPDH | Reverse | GAAGATGGTGATGGGATTTC |
| U6 | Forward | CTCGCTTCGGCAGCACA |
| U6 | Reverse | AACGCTTCACGAATTTGCGT |
| 18s RNA | Forward | CAGCCACCCGAGATTGAGCA |
| 18s RNA | Reverse | TAGTAGCGACGGGCGGTGTG |
| β-actin | Forward | CTCCATCCTGGCCTCGCTGT |
| β-actin | Reverse | GCTGTCACCTTCACCGTTCC |
| PIK3R1 | Forward | AAGAAGTTGAACGAGTGGTTGG |
| PIK3R1 | Reverse | GCCCTGTTTACTGCTCTCCC |
| CHRM3 | Forward | GTAGCCAGCAATGCCTCTG |
| CHRM3 | Reverse | CAGGAGCCCAAAGGACAAAG |
| HIPK2 | Forward | CTGTTTGTGTGGTGGTGTTG |
| HIPK2 | Reverse | GAGAGATTCGTTTGGAGATTGG |
| MAFB | Forward | AGACAGGCTTTGCGTCCTAA |
| MAFB | Reverse | CGTTAGTTGCCAATGTATGG |
| hsa-miR-3688-3p | Forward | TATGGAAAGACTTTGTTACTCT |
| hsa-miR-508-5p | Forward | TACTCCAGAGGGCGTCACTCATG |
| hsa-miR-647 | Forward | GTGGCTGCACTCACTTCCTTC |
| hsa-miR-532-3p | Forward | CCTCCCACACCCAAGGCTTGCA |
| hsa-miR-345-3p | Forward | GCCCTGAACGAGGGGTCTGGAG |
| hsa-miR-6824-3p | Forward | TCTCTGGTCTTGCCACCCCAG |
| hsa-miR-7152-3p | Forward | TCTGGTCCTGGACAGGAGGC |
| hsa-miR-198 | Forward | GGTCCAGAGGGGAGATAGGTTC |
| hsa-miR-4691-5p | Forward | GTCCTCCAGGCCATGAGCTGCGG |
| hsa-miR-767-5p | Forward | TGCACCATGGTTGTCTGAGCATG |
| hsa-miR-4758-3p | Forward | TGCCCCACCTGCTGACCACCCTC |
| Universal 5’ primer |  | GCGAGCACAGAATTAATACGAC |

**Table S3**

| **siRNAs** |  |
| --- | --- |
| circAKT3 si circ-1 | AUGGAAAAACAGCUUUUUAUC |
| circAKT3 si circ-2 | AAACACCUUUUUAUCAUAUAC |
| si PIK3R1 | UUGUUGGCUCACAGUAGUGG |
| si nc | UUCUCCGAACGUGUCACGUTT |

**Table S4**

| **mimics and inhibitors** |  |
| --- | --- |
| mimics miR-198 | GGUCCAGAGGGGAGAUAGGUUC |
| mimics nc | UUCUCCGAACGUGUCACGUTT |
| anti-miR-198 | GAACCUACUCCCCUCUCUCCACC |
| anti-nc | UUCUCCGAACGUGUCACGUTT |

**Table S5**

| **Biotinylated probes** |  |
| --- | --- |
| circAKT3 | Biotin-TTTTCCATTTGTCGAGAGAGCGGGTGTTCTCTGAGG  ACCGCACACG |
| Olige probe | Biotin-AAACAGTACTGGTGTGTAGTACGAGCTGAAGCTAC |

**Table S6**

| **FISH probes** |  |
| --- | --- |
| circAKT3 | Biotin-TTTGTCGAGAGAGCGGGTGTTCTCTGAGGACCGCACACG  TTTCTA |
| miR-198 | Digoxin-GAACCTACTCCCCTCTCTCCACC |

**Table S7**

| Antibody | Supplier | Catalogue | Primary/secondary | Host |
| --- | --- | --- | --- | --- |
| Caspase-3 | CST | 9665 | P | Rabbit |
| Cleaved caspase-3 | CST | 9664 | P | Rabbit |
| γH2AX | Abcam | ab81299 | P | Rabbit |
| BRCA1 | Abcam | ab191042 | P | Rabbit |
| PIK3R1 | Abcam | ab86714 | P | Mouse |
| p110α | CST | 4255 | P | Rabbit |
| AKT | CST | 4685 | P | Rabbit |
| p-AKT | CST | 4060 | P | Rabbit |
| GAPDH | Abcam | ab8245 | P | Mouse |
| Goat anti-rabbit IgG | Abcam | ab6721 | S | Goat |
| Goat anti-mouse IgG | Abcam | ab6789 | S | Goat |

**Table S8**

| circRNA ID | Location | Genomic length(bp) | Spliced length(bp) |
| --- | --- | --- | --- |
| hsa_circ_0026129 | Chr12:49578582-49579773 | 1191 | 1191 |
| hsa_circ_0073206 | Chr5:80733248-80946158 | 212910 | 895 |
| hsa_circ_0017247 | Chr1:243736227-243859018 | 122791 | 773 |
| hsa_circ_0071966 | Chr5:14601073-14601551 | 478 | 284 |
| hsa_circ_0073205 | Chr5:80733248-80809536 | 76288 | 675 |
| circAKT3（hsa_circ_0000199） | Chr1:243708811-243736350 | 27539 | 555 |
| hsa_circ_0073212 | Chr5:80756873-80911376 | 154503 | 490 |
| hsa_circ_0015899 | Chr1:201755556-201796102 | 40546 | 9830 |
| hsa_circ_0028122 | Chr12:109654422-109694041 | 39619 | 2913 |
| hsa_circ_0078397 | Chr6:157150360-157522622 | 372262 | 3352 |
| hsa_circ_0088236 | Chr9:119065035-119106990 | 41955 | 827 |
| hsa_circ_0076194 | Chr6:36651873-36655116 | 3243 | 2039 |
| hsa_circ_0021762 | Chr11:36654833-36669705 | 14872 | 362 |
| hsa_circ_0003825 | Chr7:22999874-23015924 | 16050 | 461 |
| hsa_circ_0043393 | Chr17:37339965-37340636 | 671 | 321 |
| hsa_circ_0082666 | Chr7:139246315-139305309 | 58994 | 13335 |
| hsa_circ_0082675 | Chr7:139299031-139305309 | 6278 | 371 |
| hsa_circ_0017540 | Chr10:5037510-5042858 | 5348 | 594 |
| hsa_circ_0058135 | Chr2:216274656-216289048 | 14392 | 1086 |
| hsa_circ_0058147 | Chr2:216283964-216286966 | 3002 | 426 |

**Table S9**

|  | Univariate analysis | | | Multivariate analysis | | |
| --- | --- | --- | --- | --- | --- | --- |
|  | HR | 95% CI | p value | HR | 95% CI | p value |
| **Age（years）** |  |  |  |  |  |  |
| < 60 | 1.000 |  |  |  |  |  |
| ≥60 | 0.759 | 0.496-1.162 | 0.204 |  |  |  |
| **Gender** |  |  |  |  |  |  |
| Male | 1.000 |  |  |  |  |  |
| Female | 0.974 | 0.597-1.589 | 0.915 |  |  |  |
| **Tumor size(cm)** |  |  |  |  |  |  |
| < 3.5 | 1.000 |  |  | 1.000 |  |  |
| ≥3.5 | 1.648 | 1.073-2.531 | 0.023 | 1.643 | 1.054-2.560 | 0.028 |
| **Histological grade** |  |  |  |  |  |  |
| Well-moderately | 1.000 |  |  | 1.000 |  |  |
| Poorly-signet | 1.662 | 1.037-2.664 | 0.035 | 1.382 | 0.856-2.233 | 0.186 |
| **Clinical stage** |  |  |  |  |  |  |
| II | 1.000 |  |  | 1.000 |  |  |
| III | 2.044 | 1.285-3.250 | 0.003 | 1.770 | 1.104-2.838 | 0.018 |
| **circAKT3 expression** |  |  |  |  |  |  |
| Low | 1.000 |  |  | 1.000 |  |  |
| High | 1.585 | 1.044-2.408 | 0.031 | 1.599 | 1.039-2.461 | 0.033 |
