# Supplementary figures and images for: Circular RNA AKT3 upregulates PIK3R1 to enhance cisplatin resistance in gastric cancer via miR-198 suppression
Source: Mol Cancer. 2019 Mar 30;18:71. doi: 10.1186/s12943-019-0969-3 (PMC6441201; doi:10.1186/s12943-019-0969-3)

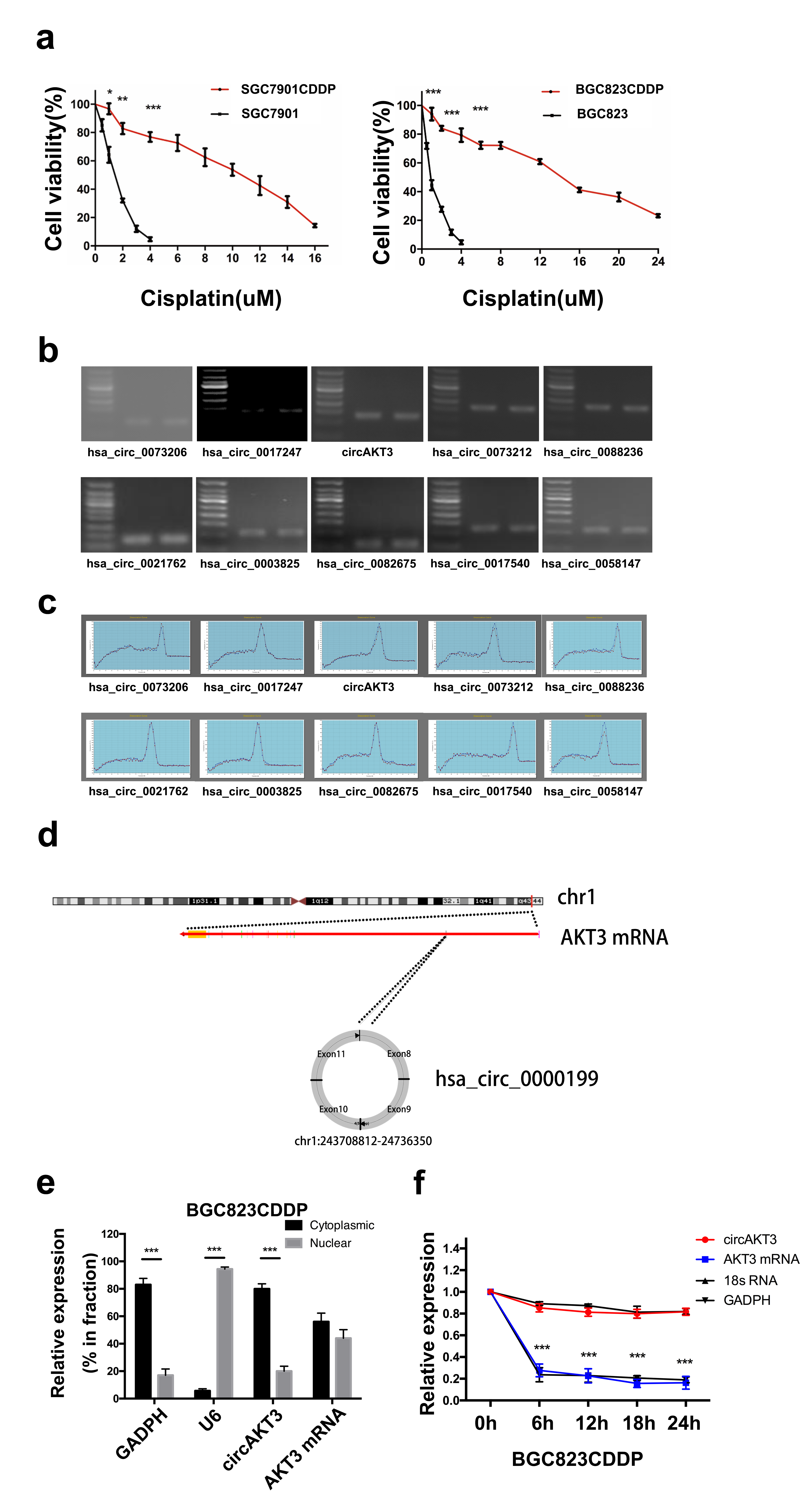

Supplement: Supplementary file 2 — Figure S1. a SGC7901CDDP/SGC7901 and BGC823CDDP/BGC823 cell viability in response to different concentrations of cisplatin. b RT-PCR products with divergent primers showing a single, distinct product of the expected size. c Melting curves of RT-qPCR product of verified circRNAs, indicating the specificity of RT-qPCR products with no primer dimers or nonspecific amplified products. d Schematic illustrating that circAKT3 (hsa_circ_0000199) is derived from exons 8, 9, 10, and 11 of the AKT3 gene (555 bp). e Levels of small nucleolar RNA (U6, as a positive control for the nuclear fraction), GAPDH (positive control for the cytoplasmic fraction), AKT3 mRNA and circRNAs from nuclear and cytoplasmic fractions of BGC823CDDP cells. f RNA stability of the circular and linear transcripts of AKT3 and 18S rRNA in BGC823CDDP cells. The results are presented as the mean ± SEM. *P<0.05, **P<0.01, ***P<0.001. (TIF 2497 kb) [file 12943_2019_969_MOESM2_ESM.tif]

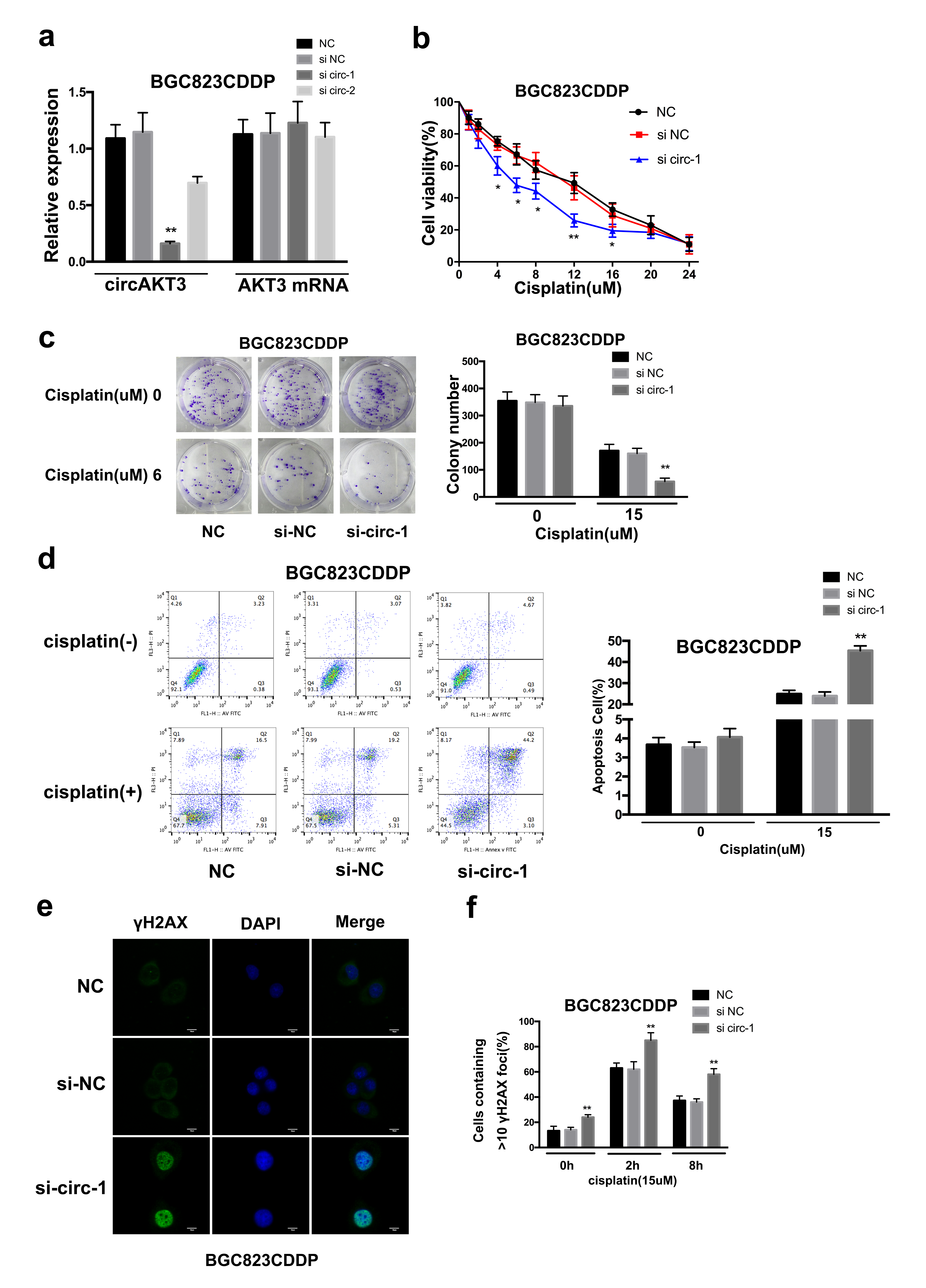

Supplement: Supplementary file 4 — Figure S2. a RT-qPCR results for the circular and linear transcripts of AKT3 in BGC823CDDP cells treated with or without siRNA (NC, negative control; si-NC, control oligonucleotides with scramble sequence; si-circ-1 and si-circ-2, oligonucleotides targeting the back-splice junction). b Relative cell viability of NC BGC823CDDP cells and BGC823CDDP cells transfected with si-NC- or si-circ-1 after CDDP treatment at the indicated concentrations for 48 h. c Colony-forming ability of the NC BGC823CDDP cells and si-NC- or si-circ-1-transfected BGC823CDDP cells in the absence (Vehicle) or presence of CDDP (15 μM) for 48 h. d The apoptosis rates of NC BGC823CDDP cells and BGC823CDDP cells transfected with si-NC or si-circ-1 in the absence (Vehicle) or upon CDDP (15 μM) for 48 h by flow cytometry. e Immunofluorescence staining of γH2AX foci in NC BGC823CDDP cells and BGC823CDDP cells transfected with si-NC or si-circ-1 at 2 h after CDDP treatment (15 μM). Scale bars, 10 μm. f Percentage of cells containing >10 γH2AX foci in NC BGC823CDDP cells and BGC823CDDP cells transfected with si-NC or si-circ-1 at 0 to 8 h after CDDP treatment (15 μM) removal. The results are presented as the mean ± SEM. *P<0.05, **P<0.01, ***P<0.001. (TIF 5287 kb) [file 12943_2019_969_MOESM4_ESM.tif]

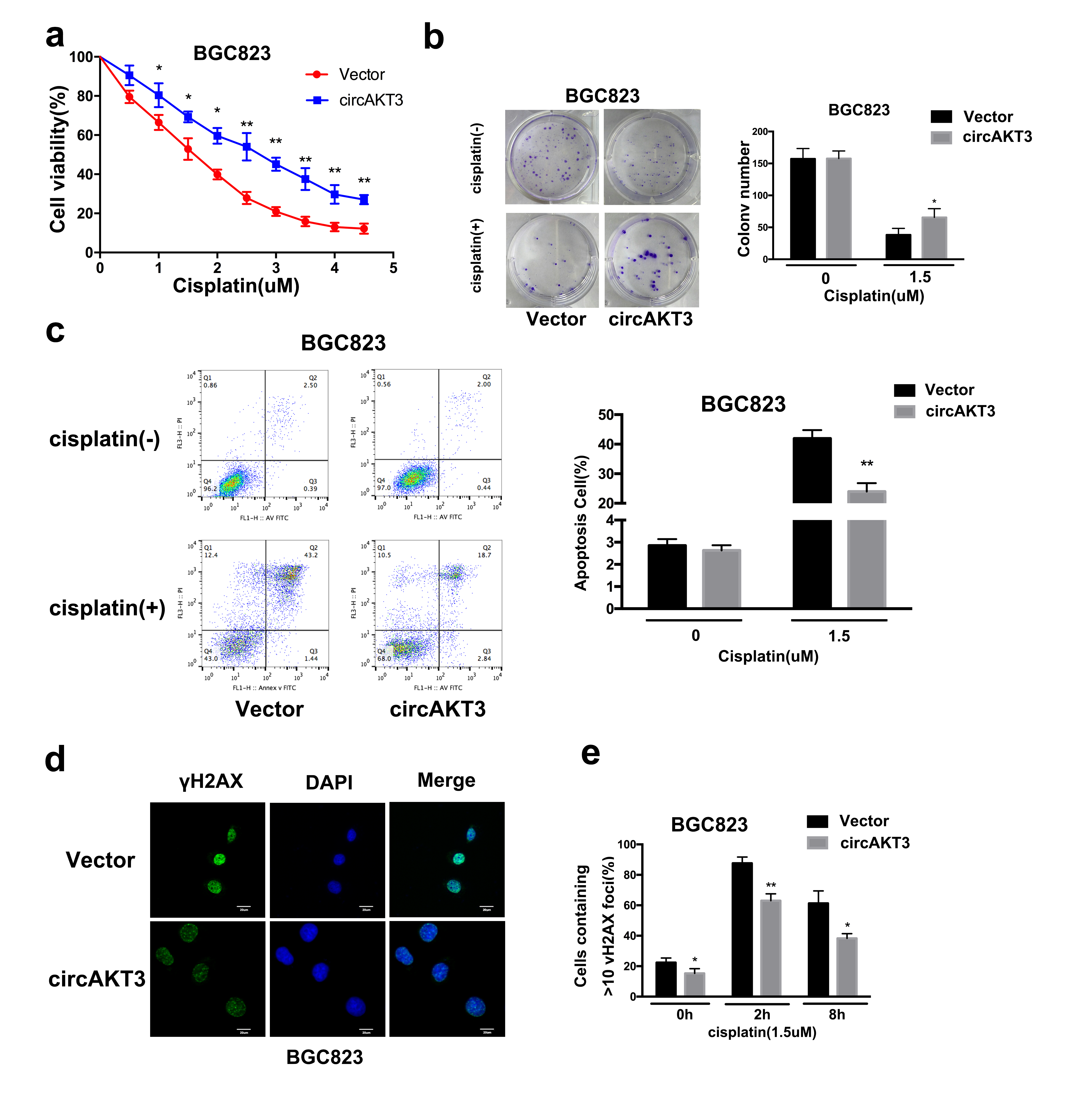

Supplement: Supplementary file 5 — Figure S3. a Relative cell viability of circAKT3- or vector-transfected BGC823 cells with CDDP treatment at the indicated concentrations for 48 h. b Colony-forming ability of BGC823 cells transfected with circAKT3 or vector in the absence (Vehicle) or presence of CDDP (1.5 μM) for 48 h. c The apoptosis rates of BGC823 cells transfected with circAKT3 or vector upon CDDP (1.5 μM) for 48 h by flow cytometry. d Immunofluorescence staining of γH2AX foci in BGC823 cells transfected with circAKT3 or vector at 2 h after CDDP treatment (1.5 μM). Scale bars, 10 μm. e Percentage of cells containing >10 γH2AX foci in BGC823 cells transfected with circAKT3 or vector at 0 to 8 h after CDDP treatment (1.5 μM) removal. The results are presented as the mean ± SEM. *P<0.05, **P<0.01, ***P<0.001. (TIF 3594 kb) [file 12943_2019_969_MOESM5_ESM.tif]

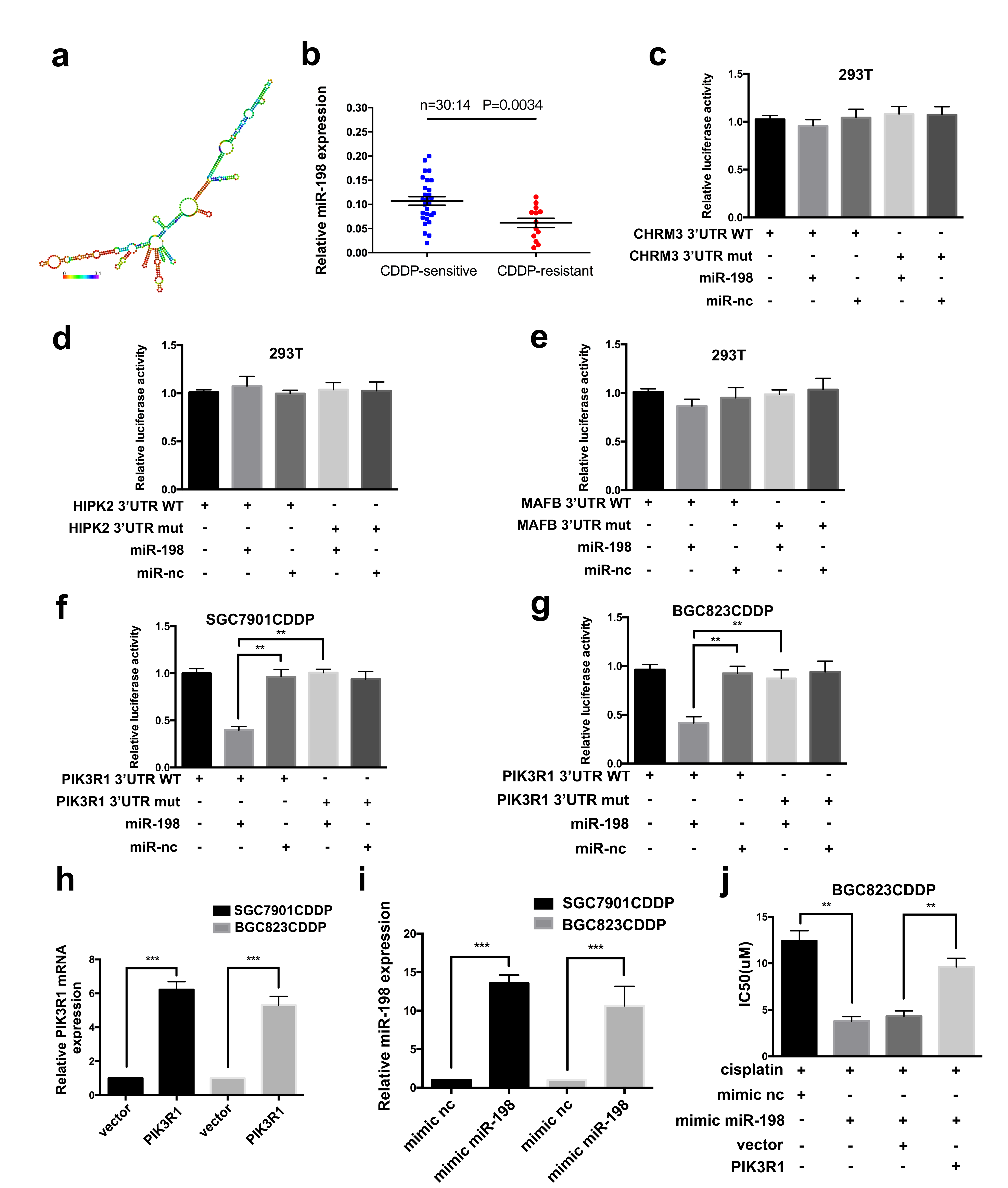

Supplement: Supplementary file 6 — Figure S4. a Predicted secondary structure of circAKT3 using the Vienna RNA package. b The expression of miR-198 was analyzed using RT-qPCR in tissues of cohort 1. c, d, e The relative luciferase activities were analyzed in 293T cells cotransfected with miR-198 mimics or miR-NC and luciferase reporter vectors containing the WT or Mut 3’UTR of CHRM3 (C), HIPK2 (D), and MAFB (E). f & g The relative luciferase activities were analyzed in SGC7901CDDP(f) and BGC823CDDP(g) cells cotransfected with miR-198 mimics or miR-NC and luciferase reporter vectors PIK3R1 3’UTR (WT) or PIK3R1 3’UTR (Mut). h & i The expression levels of PIK3R1 (h) and miR-198 (i) in SGC7901CDDP and BGC823CDDP cells after transfection of PIK3R1 plasmids or miR-198 mimics were detected by RT-qPCR. j The IC50 of miR-198 was analyzed by the CCK8 assay. BGC823CDDP cells were transfected with miR-198 mimic alone or cotransfected with the indicated vectors upon CDDP exposure (15 μM) for 48 h. The results are presented as the mean ± SEM. *P<0.05, **P<0.01, ***P<0.001. (TIF 1650 kb) [file 12943_2019_969_MOESM6_ESM.tif]

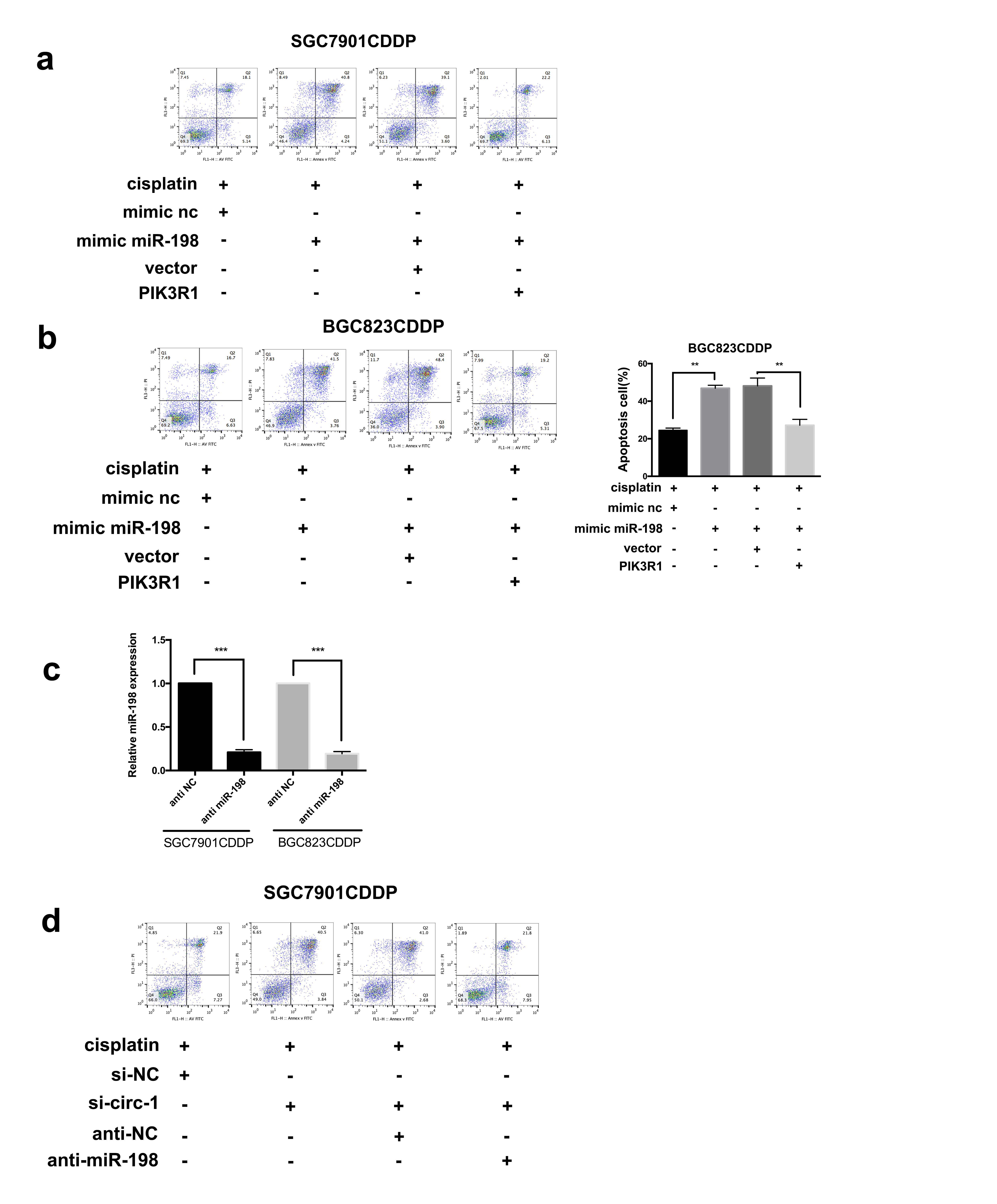

Supplement: Supplementary file 7 — Figure S5. a Apoptotic flow cytometry. SGC7901CDDP cells were transfected with miR-198 mimic alone or cotransfected with the indicated vectors upon CDDP exposure (6 μM) for 48 h b The apoptosis rate was analyzed by flow cytometry. BGC823CDDP cells were transfected with miR-198 mimic alone or cotransfected with the indicated vectors upon CDDP exposure (15 μM) for 48 h. c The expression levels of miR-198 in SGC7901CDDP and BGC823CDDP cells after transfection of anti-miR-198 were detected by RT-qPCR. d Apoptotic flow cytometry. SGC7901CDDP cells transfected with indicated vectors alone or cotransfected the inhibitors upon CDDP exposure (6 μM) for 48 h. The results are presented as the mean ± SEM. *P<0.05, **P<0.01, ***P<0.001. (TIF 2595 kb) [file 12943_2019_969_MOESM7_ESM.tif]

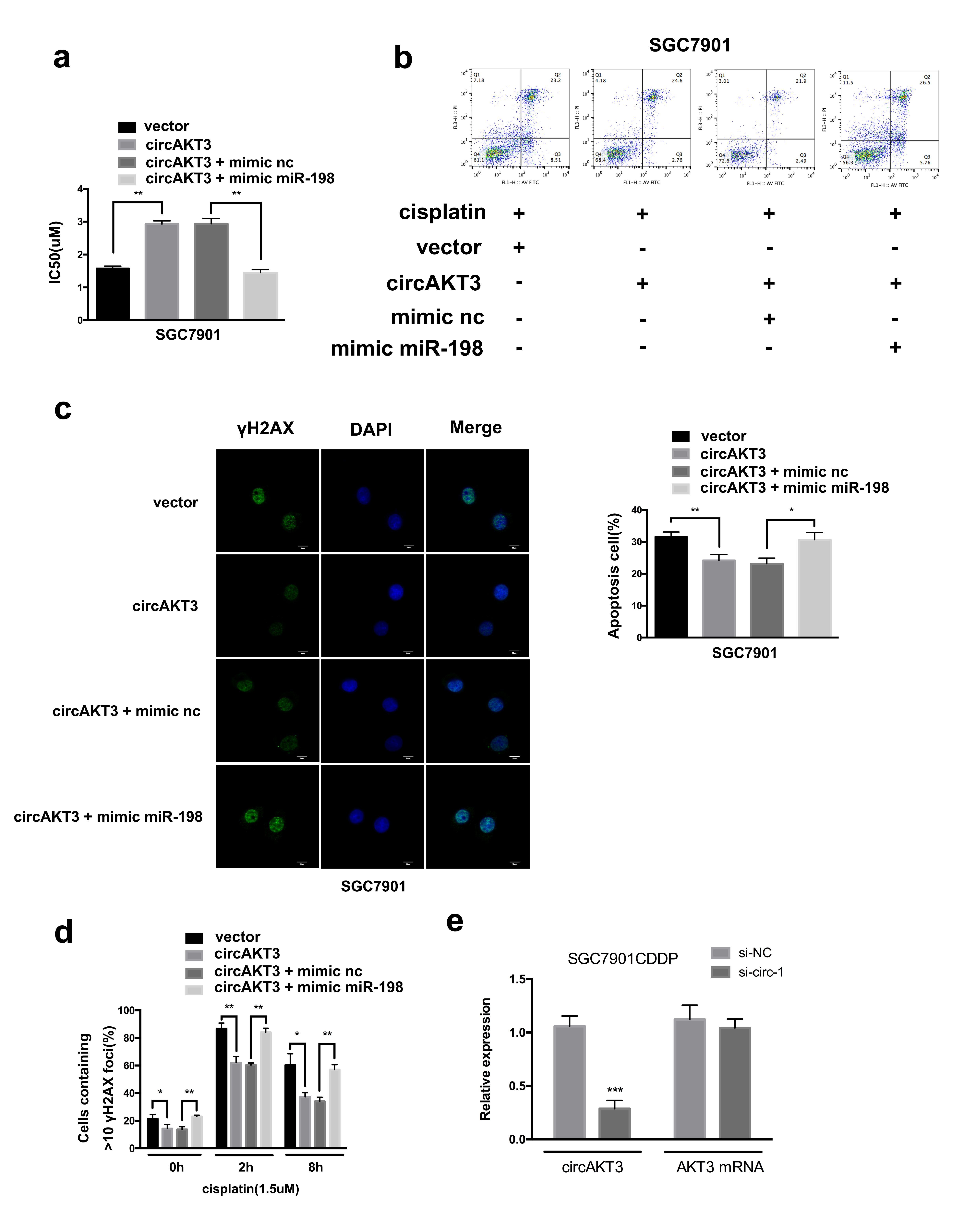

Supplement: Supplementary file 8 — Figure S6. a The IC50 was analyzed by CCK8 assay, SGC7901 cells were transfected with inhibitor alone or cotransfected with the indicated vectors upon CDDP exposure (1.5 μM) for 48 h. b The apoptosis rate was analyzed by flow cytometry. SGC7901 cells were transfected with inhibitor alone or cotransfected with the indicated vectors upon CDDP exposure (1.5 μM) for 48 h. c Immunofluorescence staining of γH2AX foci in SGC7901 cells transfected with inhibitor alone or cotransfected with the indicated vectors at 2 h after CDDP treatment (1.5 μM). Scale bars =10 μm. d Percentage of cells containing >10 γH2AX foci in SGC7901 cells transfected with inhibitor alone or cotransfected with the indicated vectors at 0 to 8 after CDDP treatment (1.5 μM) removal. e SGC7901CDDP cells stably expressing circAKT3 siRNA (si-circ-1) and its negative control siRNA (si-NC) were generated by infection with lentiviruses, the expression levels of cricAKT3 were detected by RT-qPCR. The results are presented as the mean ± SEM. *P<0.05, **P<0.01, ***P<0.001. (TIF 2160 kb) [file 12943_2019_969_MOESM8_ESM.tif]

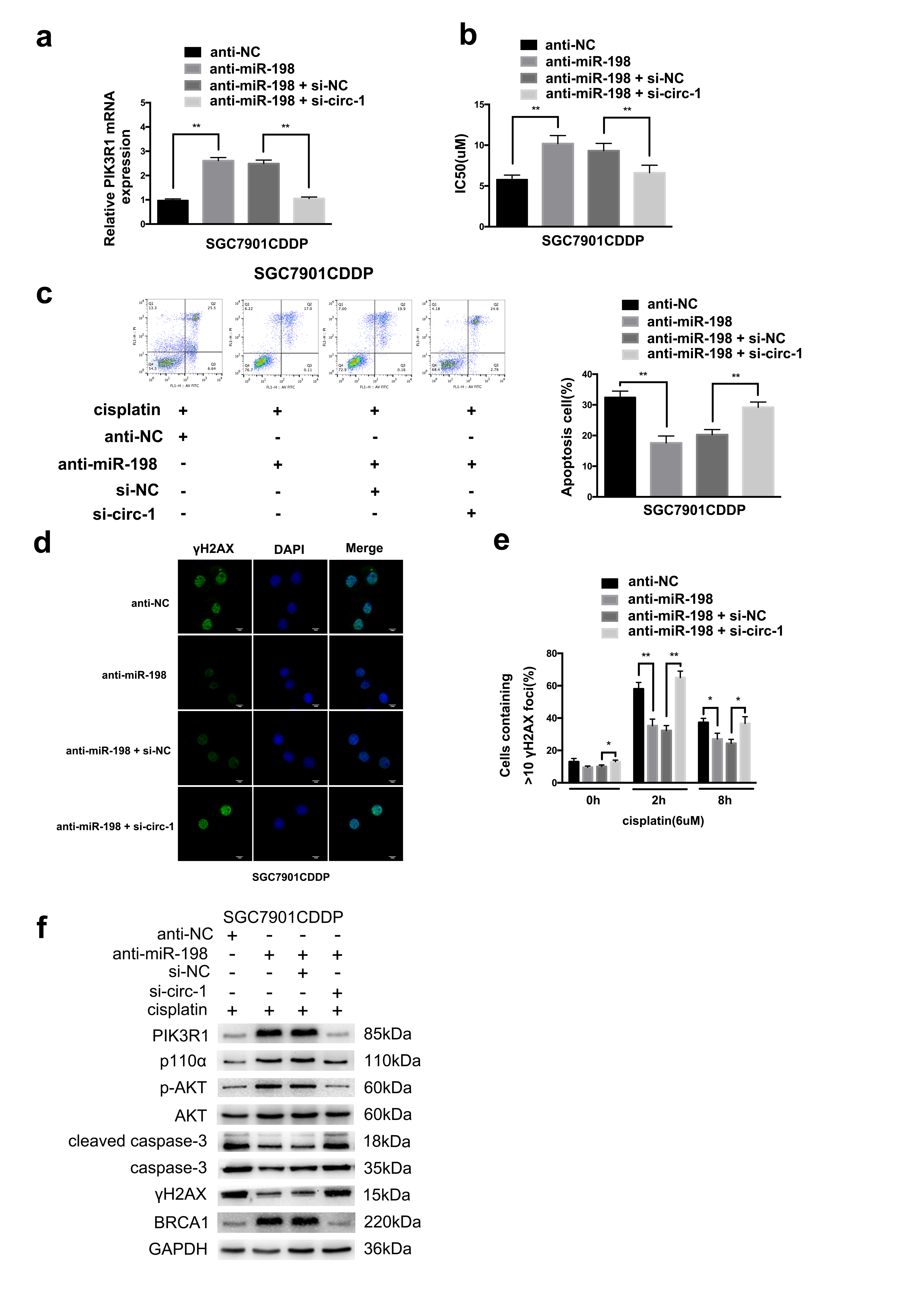

Supplement: Supplementary file 9 — Figure S7. a The expression levels of PIK3R1 were analyzed using RT-qPCR. SGC7901CDDP cells were cotransfected with inhibitors alone or the indicated vectors. b The IC50 of circAKT3 was analyzed by the CCK8 assay. SGC7901CDDP cells were transfected with inhibitor alone or cotransfected with the indicated vectors upon CDDP exposure (6 μM) for 48 h. c The apoptosis rates of SGC7901CDDP cells transfected with inhibitor alone or cotransfected with the indicated vectors upon CDDP exposure (6 μM) for 48 h. d Immunofluorescence staining of γH2AX foci in SGC7901CDDP cells transfected with inhibitor alone or cotransfected with the indicated vectors at 2 h after CDDP treatment (6 μM). Scale bars, 10 μm. e Percentage of cells containing >10 γH2AX foci in SGC7901CDDP cells transfected with inhibitor alone or cotransfected with the indicated vectors at 0 to 8 after CDDP treatment (6 μM) removal. f The expression levels of PIK3R1, apoptosis markers, γH2AX, BRCA1 and PI3K/AKT signaling molecules were determined using western blotting in SGC7901CDDP cells transfected with inhibitor alone or cotransfected with the indicated vectors after CDDP treatment (6 μM). The results are presented as the mean ± SEM. *P<0.05, **P<0.01, ***P<0.001. (TIF 2258 kb) [file 12943_2019_969_MOESM9_ESM.tif]

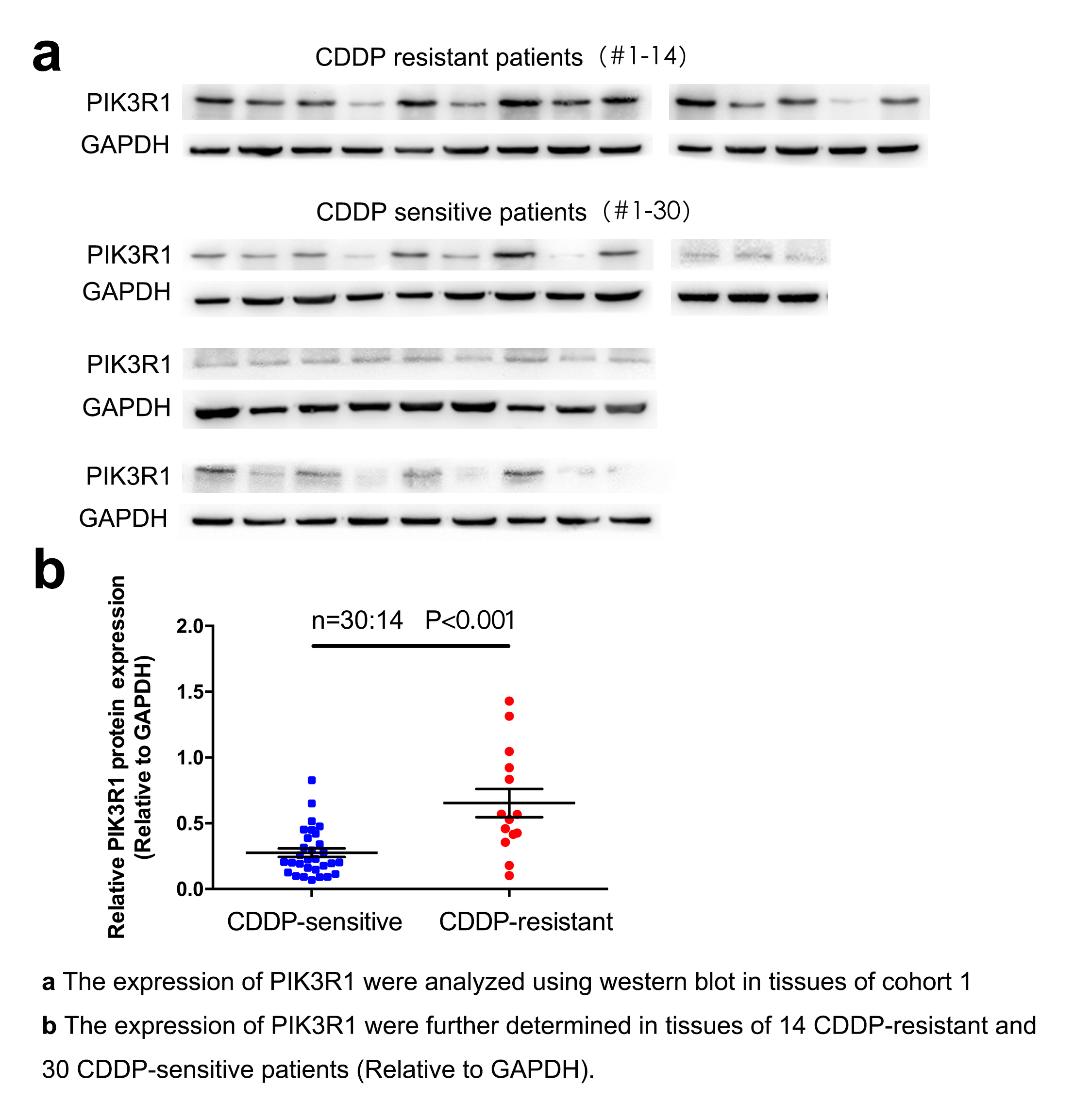

Supplement: Supplementary file 10 — Figure S8. a The expression of PIK3R1 were analyzed using western blot in tissue of cohort 1. b The expression of PIK3R1 were further determined in tissues of 14 CDDP-resistant and 30 CDDP-sensitive patients (Relative to GAPDH). (TIF 604 kb) [file 12943_2019_969_MOESM10_ESM.tif]
