# Supplementary material for: Circular RNA AKT3 upregulates PIK3R1 to enhance cisplatin resistance in gastric cancer via miR-198 suppression
Source: Mol Cancer. 2019 Mar 30;18:71. doi: 10.1186/s12943-019-0969-3 (PMC6441201; doi:10.1186/s12943-019-0969-3)
Supplement: Supplementary file 3 — Additional Dataset (1) miRNA target prediction of circAKT3. Additional Dataset (2) Eight miR-198 binding sites mutation of circAKT3. Additional Dataset (3) mRNA target prediction of miR-198. (ZIP 1800 kb) [file 12943_2019_969_MOESM3_ESM.zip › Additional Dataset 2.docx]

 Additional Dataset 2, Eight miR-198 binding sites mutation of circAKT3

target: hsa_circ_0000199|NM_181690|AKT3

length: 555

miRNA : hsa-miR-198

length: 22

mfe: -29.5 kcal/mol

p-value: 1.000000e+00

position 21

target 5' A GG G GA G 3'

GAGC GU UUCUCU GGACC

CUUG UA AGGGGA CCUGG

miRNA 3' GA G GA 5'

------------------------------------------------------------

dataset: 1

target: hsa_circ_0000199|NM_181690|AKT3

length: 555

miRNA : hsa-miR-198

length: 22

mfe: -27.6 kcal/mol

p-value: 1.000000e+00

position 505

target 5' A A A G A 3'

GAC C GUUUCUUCUCUGGA U

UUG G UAGAGGGGAGACCU G

miRNA 3' C A G 5'

------------------------------------------------------------

dataset: 1

target: hsa_circ_0000199|NM_181690|AKT3

length: 555

miRNA : hsa-miR-198

length: 22

mfe: -27.1 kcal/mol

p-value: 1.000000e+00

position 63

target 5' A A G C A 3'

GAA UUGUCUCU C UUGGACU

CUU GAUAGAGG G GACCUGG

miRNA 3' G G A 5'

------------------------------------------------------------

dataset: 1

target: hsa_circ_0000199|NM_181690|AKT3

length: 555

miRNA : hsa-miR-198

length: 22

mfe: -24.7 kcal/mol

p-value: 1.000000e+00

position 220

target 5' U GGCA AGAAUA C A 3'

UCUGU CUCC UCUGG ACC

GGAUA GAGG AGACC UGG

miRNA 3' CUU GG 5'

------------------------------------------------------------

dataset: 1

target: hsa_circ_0000199|NM_181690|AKT3

length: 555

miRNA : hsa-miR-198

length: 22

mfe: -24.0 kcal/mol

p-value: 1.000000e+00

position 335

target 5' U U ACAA A A 3'

ACCU UCU CC GGACC

UGGA AGA GG CCUGG

miRNA 3' CU U G GAGA 5'

------------------------------------------------------------

dataset: 1

target: hsa_circ_0000199|NM_181690|AKT3

length: 555

miRNA : hsa-miR-198

length: 22

mfe: -23.1 kcal/mol

p-value: 1.000000e+00

position 109

target 5' U GUG AAGUUGGAGAA AAUG A 3'

ACC AUCUC UCU CUGGAC

UGG UAGAG GGG GACCUG

miRNA 3' CU A A G 5'

------------------------------------------------------------

dataset: 1

target: hsa_circ_0000199|NM_181690|AKT3

length: 555

miRNA : hsa-miR-198

length: 22

mfe: -20.1 kcal/mol

p-value: 1.000000e+00

position 403

target 5' C A A G 3'

GAAC CUCUCUUC GAU

CUUG GAGGGGAG CUG

miRNA 3' GAUA AC G 5'

------------------------------------------------------------

dataset: 1

target: hsa_circ_0000199|NM_181690|AKT3

length: 555

miRNA : hsa-miR-198

length: 22

mfe: -20.0 kcal/mol

p-value: 1.000000e+00

position 452

target 5' A CAA AAA G GGUGGA A 3'

GGAUC AU C CCUU GGACC

CUUGG UA G GGAG CCUGG

miRNA 3' A GA G A 5'

------------------------------------------------------------
